# Eco-Spatial Modeling of Two Giant Flying Squirrels (Sciuridae: *Petaurista*): Navigating Climate Resilience and Conservation Roadmap in the Eastern Himalaya and Indo-Burma Biodiversity Hotspots

**DOI:** 10.3390/life15040589

**Published:** 2025-04-03

**Authors:** Imon Abedin, Manokaran Kamalakannan, Tanoy Mukherjee, Hilloljyoti Singha, Dhriti Banerjee, Hyun-Woo Kim, Shantanu Kundu

**Affiliations:** 1 Department of Zoology, Bodoland University, Kokrajhar 783370, India; 2 Mammal and Osteology Section, Zoological Survey of India, Kolkata 700053, India; 3 Agricultural and Ecological Research Unit, Indian Statistical Institute, Kolkata 700108, India; 4 Centre for Wildlife Research and Biodiversity Conservation, Bodoland University, Kokrajhar 783370, India; 5 Zoological Survey of India, Prani Vigyan Bhawan, Kolkata 700053, India; 6 Department of Marine Biology, Pukyong National University, Busan 48513, Republic of Korea; 7 Marine Integrated Biomedical Technology Center, National Key Research Institutes in Universities, Pukyong National University, Busan 48513, Republic of Korea; 8 Department of Biology, Faculty of Science and Technology, Airlangga University, Surabaya 60115, Indonesia; 9 Ocean and Fisheries Development International Cooperation Institute, College of Fisheries Science, Pukyong National University, Busan 48513, Republic of Korea; 10 International Graduate Program of Fisheries Science, Pukyong National University, Busan 48513, Republic of Korea

**Keywords:** biodiversity conservation, biological corridor connectivity, habitat fragmentation, species distribution model, species management plan, transboundary region

## Abstract

Global warming and anthropogenic threats are significant drivers of biodiversity loss, particularly impacting smaller mammalian species. Hence, this study assessed two overlooked giant flying squirrel species, *Petaurista magnificus* and *Petaurista nobilis*, distributed across the transboundary regions of the Eastern Himalayas and Indo-Burma biodiversity hotspots. Utilizing a maximum entropy (MaxEnt) species distribution model, this study delineated suitable habitats within the IUCN-defined extent of both *Petaurista* species based on two modeling approaches: the habitat–climate model (HCM) and the climate-only model (COM). The models identified suitable habitat coverage of only 3.92% (HCM) and 3.75% (COM) for *P. magnificus* and 14.17% (HCM) and 10.04% (COM) for *P. nobilis*. However, as the HCM integrates both environmental and habitat variables, providing a more holistic assessment, it revealed limited biological corridor connectivity within the IUCN-defined extent for both species. Furthermore, the future projections based on the HCM indicate habitat loss of up to 81.90% for *P. magnificus* and 89.88% for *P. nobilis* due to climate change, alongside severe fragmentation, leading to the disappearance of viable habitat patches. These remaining suitable patches are expected to shrink and become increasingly isolated in the future due to climate change. Furthermore, centroid shift analyses based on the HCM predict a northwestward shift for *P. magnificus* and a westward shift for *P. nobilis* under different climate scenarios. Hence, to address these conservation challenges, the study underscores the necessity for extensive field surveys, genetic assessments, habitat corridor evaluations, and the establishment of transboundary conservation frameworks to formulate an evidence-based species management strategy for both *Petaurista* species.

## 1. Introduction

The escalating impacts of climate change and anthropogenic pressures have placed substantial stress on natural ecosystems, which serve as critical habitats for a diverse assemblage of mammalian species [1,2]. These ecosystems are fundamental to species survival, facilitating ecological interactions and sustaining viable populations over time [3]. However, the rapid degradation of these habitats has led to significant disruptions in global ecological stability [4,5]. Consequently, approximately half a million species are now at risk of extinction, contributing to the ongoing sixth mass extinction event [6,7,8]. Given the severity of this biodiversity crisis, prioritizing targeted research and conservation initiatives is imperative [3,9,10]. Although numerous studies have attempted to address these challenges, most have disproportionately focused on charismatic megafauna, leaving many less conspicuous species understudied and poorly characterized [10,11,12,13]. Addressing this knowledge gap necessitates an integrative approach that combines species distribution modeling (SDM) with the identification of key ecological drivers shaping spatial patterns and population dynamics [14]. Advancing these methodologies enhances the understanding of species ecology and also informs more effective conservation strategies under this habitat loss scenario.

Giant flying squirrels are a distinct group of rodents that have received limited and sporadic scientific attention despite their significant ecological role [15]. These mammals are known to contribute to forest ecosystem functioning by providing essential ecosystem services such as pollination and seed dispersal, and serve as indicators of ecosystem health [16,17]. They are uniquely adapted for gliding, possessing a specialized membrane, or “patagium”, that extends between their limbs, enabling aerial locomotion. Globally, 57 species of flying squirrels, classified under 15 genera and forming a monophyletic group within the tribe Pteromyini, subfamily Sciurinae, and family Sciuridae, are widely distributed across Eurasia and North America [18,19]. Among them, 19 species are classified under the genus *Petaurista* and are exclusively distributed across the Asian continent [20]. Their gliding ability is considered an adaptive extension of their arboreal lifestyle, facilitating efficient resource exploitation and predator evasion within forested environments [21,22]. This specialized mode of locomotion has likely played a crucial role in the diversification of vertebrate lineages that exhibit gliding behavior by allowing access to tall canopies and expanding ecological opportunities [23,24]. Despite their ecological significance, flying squirrel populations have been experiencing a steady decline over the last few decades, primarily due to habitat loss driven by deforestation, degradation of primary forests, and hunting pressures, particularly in regions such as India [25,26,27,28,29]. Nearly all flying squirrel species are confined to Asia, with the highest diversity occurring in Southeast Asia, making it a global hotspot for these rodents [15]. In India, 23 species of flying squirrels have been documented, with 18 occurring in the Eastern Himalayas, a region characterized by restricted ranges and high endemism [30].

Specifically, among the 37 globally recognized species of flying squirrels, two species, viz. Hodgson’s giant flying squirrel *Petaurista magnificus* (Hodgson, 1836) and the Bhutan giant flying squirrel *Petaurista nobilis* (J. E. Gray, 1842), exhibit overlapping distributions within the transboundary landscapes of the Himalayan and Indo-Burma biodiversity hotspots [31,32]. *Petaurista magnificus* is a large flying squirrel with a head–body length of 359–490 mm and a bushy tail of 415–522 mm ending in distinct black tips. Its deep maroon fur features black and whitish grizzling, with pectoral patches ranging from saturn red with a golden tint to creamy buff. The underside varies from orange buff to chestnut orange, while the limbs and flying membranes are lighter, it has strong, sharp claws, and its hind foot measures 72–85 mm [33,34]. *P. nobilis* is a medium-sized flying squirrel with a head–body length of 347–420 mm and a tail longer than its head and body, measuring 378–510 mm. It has distinctive shoulder patches extending along the sides, forming a saddle-like pattern. The dorsal saddle is dark maroon with a narrow pale stripe, while the underside varies from salmon buff to flesh ocher. Its parachute, limbs, and sides are orange rufous or orange buff. The hind foot measures 70.5–80.0 mm. This species was initially classified as a subspecies, *P. magnificus*, but was later recognized as a distinct species due to its unique chestnut-brown saddle surrounded by lighter golden-yellow or orange areas [35]. The most recent assessment by the IUCN SSC Small Mammal Specialist Group (SMSG) for both species was conducted nearly a decade ago, classifying *P. magnificus* as “least concern” and *P. nobilis* as “near threatened.” Given this context, it is essential to generate scientific information on their habitat preferences and responses to climate change in the current scenario. Such information will be instrumental in developing a transboundary management plan for these species and other sympatric taxa within their range. Such assessments will aid in the ecological and geographical reevaluation of both species by the IUCN SSC Small Mammal Specialist Group (SMSG), providing a foundation for facilitating broader, conservation-focused research initiatives. Enhancement of understanding the synergistic effects of climate change and land cover dynamics will provide deeper insights into the differential vulnerabilities of these species, informing more effective conservation strategies.

Hence, effective species management at both habitat and landscape levels requires a clear understanding of species distributions and the identification of suitable habitats [36]. In this context, species distribution models (SDMs) serve as valuable tools for predicting species occurrence across specific geographic regions, providing critical insights for habitat management and conservation planning [37,38,39,40]. In recent years, the integration of climatic and habitat variables into SDMs has become increasingly important for projecting species distributions, particularly in assessing range shifts in response to climate change [41,42,43,44]. It is equally essential to systematically assess and identify environmentally suitable areas that align with the species’ ecological niche and requirements, which are critical for its survival. Additionally, understanding how key environmental factors interact with changing climate conditions is essential for identifying and protecting suitable habitats. In this context, alongside identifying suitable habitats, it is equally critical to map and establish ecological corridors between these areas to facilitate connectivity, enabling metapopulations to interact, exchange genetic material, and maintain demographic stability, rather than becoming isolated and vulnerable to the risks associated with small, fragmented populations. Furthermore, identifying the directional movement of a species’ distribution center over time or across scenarios, known as centroid shift, is essential for understanding range dynamics, predicting the impacts of climate change, and guiding conservation strategies to support species persistence [43]. Furthermore, to date, only two ecological modeling and habitat suitability studies have been conducted on *Petaurista philippensis*, *Petaurista mishmiensis*, and *Petaurista mechukaensis* within the Indian subcontinent [44,45]. This represents a significant research gap concerning the ecological data of other sympatric species within the same taxonomic group. Therefore, implementing adaptive management strategies that account for future uncertainties is crucial for enhancing the resilience of these two species across their IUCN-designated range. Thus, the present study aimed to identify suitable habitats for both *P. magnificus* and *P. nobilis* within the IUCN ranges using both the habitat–climate model (HCM) and climate-only model (COM) approaches. Furthermore, since the HCM has been shown to provide a more holistic approach with a comprehensive ecological and environmental representation [46,47], further assessments were conducted using this approach exclusively. Therefore, connectivity in the current scenario, dynamics of suitable areas under future climatic conditions, habitat shape geometry and complexity, as well as centroid shifts in response to climate change, were all assessed using the HCM approach.

## 2. Materials and Methods

### 2.1. Study Area and Distribution Range

**Figure 1:**
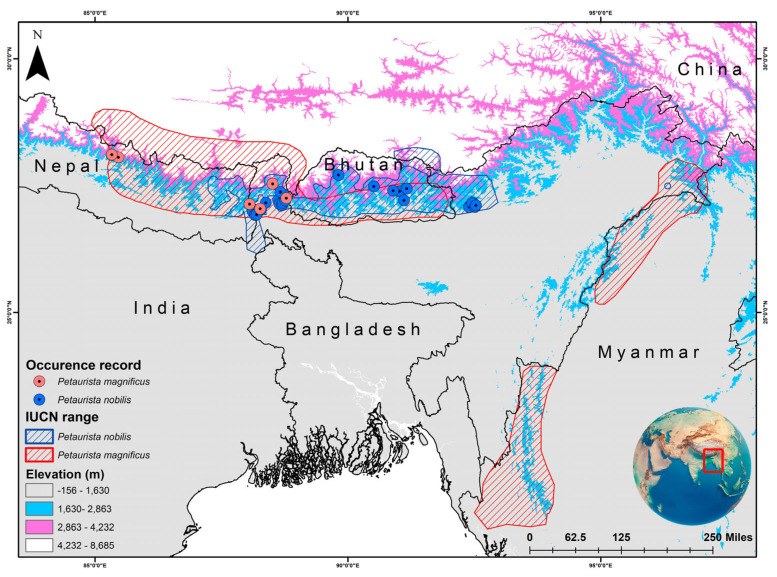
IUCN-defined distribution range of two flying squirrel species, viz. *Petaurista magnificus* and *Petaurista nobilis*, along with their presence locations obtained from secondary sources. The map was created using ArcGIS version 10.6, with the inset global map sourced from Google.

The two species have overlapping distributions within the Eastern Himalayan region, specifically across Nepal, Bhutan, and Sikkim in India [31,32]. *P. magnificus* is primarily restricted to northern South Asia, southern China, and western Southeast Asia. In South Asia, it has been recorded in Bhutan, India, and Nepal (Figure 1) [48]. In China, it is found in southern Xizang, while in Southeast Asia, its distribution is limited to western and northern Myanmar [48]. Conversely, *P. nobilis* is endemic to Bhutan, India, and Nepal (Figure 1) [49,50]. However, an observation was reported in Arunachal Pradesh, India, with subsequent records extending its range to Tawang district and suggesting its possible occurrence in Tibet, China [51,52]. Given that the IUCN SSC SMSG has established a distinct boundary for both species, the IUCN-designated range was chosen as the training area for model development and assessment. The present study primarily relied on secondary data sources for model training and execution. The occurrence records (*P. magnificus* = 22; *P. nobilis* = 36) were obtained from the GeoCAT website, which aggregates location data from GBIF and iNaturalist [53]. Additionally, locality information was collected for both species (*P. magnificus* = 6; *P. nobilis* = 9) from the museum collections archived in the National Zoological Collections of the Zoological Survey of India (Table S1). Hence, to minimize overfitting and bias in model interpretation, records from captive and museum specimens were excluded to clearly depict their presence areas within the natural habitat. Additionally, the spatial correlation of occurrence data was analyzed at a resolution of 1 km^2^ using SDM Toolbox v2.4 [54]. This resolution was selected to align with the pixel size of raster data, thereby reducing overfitting and enhancing model accuracy.

### 2.2. Habitat Requirements

The two *Petaurista* species are found in two major biodiversity hotspots, the Eastern Himalayas and Indo-Burma, each with diverse ecosystems and climates [55]. The Eastern Himalayas, spanning parts of India, Bhutan, Nepal, and Myanmar, include forests ranging from tropical lowlands to alpine meadows, with temperatures from −30 °C to 35 °C and rainfall up to 5000 mm. Indo-Burma, covering northeastern India, Myanmar, and Southeast Asia, features tropical rainforests, wetlands, and montane forests, with temperatures from 5 °C to 40 °C and rainfall reaching 4000 mm. Specifically, *P. magnificus* is found in patches of evergreen forests, occurring at elevations ranging from 400 m above sea level (a.s.l.) in Arunachal Pradesh, India, to 3700 m a.s.l. in Nepal [30]. In contrast, *P. nobilis* primarily inhabits montane forests, with a distribution spanning an elevation gradient of 1500 to 3000 m a.s.l. [32].

### 2.3. Selection of Variables

A combination of bioclimatic, topographic, habitat, and anthropogenic variables was utilized to identify suitable habitat patches for the flying squirrels within the study area [56]. The standard set of 19 bioclimatic variables was obtained from the WorldClim database (https://www.worldclim.org/) and extracted for use within the IUCN-designated ranges of both species [57]. Given that these species primarily inhabit evergreen and montane forests, the Euclidean distance to evergreen forests and montane forests was selected as a key habitat variable for *P. magnificus* and *P. nobilis*, respectively. The evergreen forests are characterized by dense, year-round green foliage in tropical/subtropical regions, while montane forests are altitude-dependent, with distinct vegetation zones from broadleaf trees at lower elevations to conifers and alpine meadows higher up. This raster layer was derived from Copernicus Land Use and Land Cover data and processed using the Euclidean distance function in ArcGIS v.10.8 [58]. Additionally, topographic variables, including elevation, aspect, and slope, were extracted from 90 m Shuttle Radar Topography Mission (SRTM) data (http://srtm.csi.cgiar.org/srtmdata/). The Global Human Footprint Dataset was incorporated as an anthropogenic predictor to evaluate the Human Influence Index (HII) and assess the extent of human impact on the target species [59]. All spatial variables were standardized to a resolution of 30 arcseconds (~1 km^2^) using the spatial analyst extension in ArcGIS 10.6. Moreover, to ensure analytical robustness, spatial multicollinearity testing was conducted using VisTrails software [60]. The predictor variables with a Pearson correlation coefficient (r) exceeding 0.8 were excluded from further analysis to minimize redundancy (Figures S1–S4) [61]. The final dataset included ten key variables for HCM, and seven variables for COM were used for modeling habitat suitability for the two giant flying squirrel species. Furthermore, to assess the potential impacts of climate change in HCM approach, the study analyzed future climate scenarios under two shared socioeconomic pathways (SSPs), SSP245 and SSP585, for the periods 2041–2060 and 2061–2080. The future climate projections were based on the HadGEM3-GC31 LL model, part of the Coupled Model Intercomparison Project Phase 6 (CMIP6). This model was selected for its reliability in simulating climate variability and temperature trends across South and Southeast Asia [62,63,64]. Moreover, to isolate the effects of climate change on species distribution, non-climatic variables were held constant during future climate analyses for HCM approaches, ensuring that projections remained ecologically relevant [65,66].

### 2.4. Model Development and Execution

SDM analysis was conducted using MaxEnt version 3.4.4, a widely recognized program known for its robust predictive performance in ecological modeling [67,68]. Specifically, two distinct modeling approaches were employed for both species: the habitat–climate model (HCM) and the climate-only model (COM). The HCM combines climatic and habitat raster data with topographic variables and anthropogenic factors to comprehensively assess habitat suitability [46,47,69,70]. Conversely, the COM relies solely on climatic raster data and topographic layers to analyze suitability within the training, providing a baseline for comparative scenario evaluation [43]. The model development employed a bootstrapping replication approach along with the Bernoulli generalized linear model using the ClogLog link function [71]. During this process, training data for each occurrence point were treated as *n* − 1, and model execution was evaluated over 50 replicate runs, with residual points assessed accordingly [72]. The spatial jackknife test was used to determine the influence of predictor variables on species occurrence by analyzing regularized training gain [62]. Moreover, the model evaluation was based on the area under the curve (AUC) of the receiver operating characteristic (ROC) curve [72]. The AUC values were interpreted as follows: values below 0.5 indicated insufficient predictive power, 0.5 suggested random prediction, 0.7–0.8 were considered acceptable, 0.8–0.9 were deemed excellent, and values exceeding 0.9 were classified as exceptional model performance [52,73]. Furthermore, the binary habitat suitability maps were generated using the equal test sensitivity and specificity (SES) threshold, ensuring reliable habitat predictions for the target species. The raster calculator was then applied to evaluate zonal statistics utilizing the Zonal Statistics Tool in ArcGIS 10.6 for spatial analysis [74].

### 2.5. Identification of Connectivity and Centroid Shift

Given the critical role of habitat connectivity in conservation efforts to maintain species persistence and facilitate gene flow, identifying biological connectivity within habitat patches was essential [75]. Thus, to achieve this, a circuit-based modeling approach, commonly used for designing wildlife corridors, was implemented in the HCM approach [76]. The Circuitscape toolbox in ArcGIS 10.6 was employed to simulate ecological corridors, with species occurrence points designated as nodes and the conductance surface derived from the probability outputs generated by the MaxEnt model [77]. Circuitscape models the species movement and ecological flow across landscapes by simulating connectivity pathways. In this study, the pairwise source/ground mode settings were applied, where probability maps from the MaxEnt model served as the conductance raster, and location points were used as the focal node raster within the pairwise setup module. The output was generated as current flow maps, which were further analyzed to assess connectivity patterns across the landscape. This corridor simulation was performed for both present and future climatic scenarios. Additionally, to evaluate potential distribution shifts due to climate change, the centroid displacement of suitable habitats for both flying squirrel species was analyzed across future SSP projections for both timeframes using the HCM approach. This assessment was conducted using the centroid change function within SDM Toolbox v2.4, providing insights into potential range shifts and habitat stability under changing climatic conditions [52].

### 2.6. Assessment of Habitat Shape Geometry

The qualitative and geometric characteristics of suitable habitat patches for the two flying squirrel species were analyzed under both current and projected future climatic scenarios to facilitate comparative assessments utilizing the HCM approach. This evaluation was conducted using class-level metrics in FRAGSTATS software version 4.2.1 [78], a widely recognized tool in landscape ecology and environmental management. This specific software enables the spatial analysis of habitat patterns by providing a comprehensive set of metrics and indices to assess landscape structure and composition [79]. Moreover, the key shape geometry metrics were incorporated into the analysis, including the number of patches (NPs), largest patch index (LPI), aggregate index (AI), patch density (PD), and landscape shape index (LSI) [80]. Furthermore, NPs, PD, and LPI offer detailed insights into the size and density of habitat patches within a defined geographical area. In contrast, the LSI assesses the complexity of patch shapes, while the AI quantifies the proximity and clustering of patches, reflecting their degree of aggregation or dispersion across the landscape. Collectively, these metrics provide a comprehensive understanding of habitat structure, which is crucial for evaluating habitat viability under both present and future climate conditions.

## 3. Results

### 3.1. Assessment of SDM and Habitat Suitability with Corridor Connectivity

**Figure 2:**
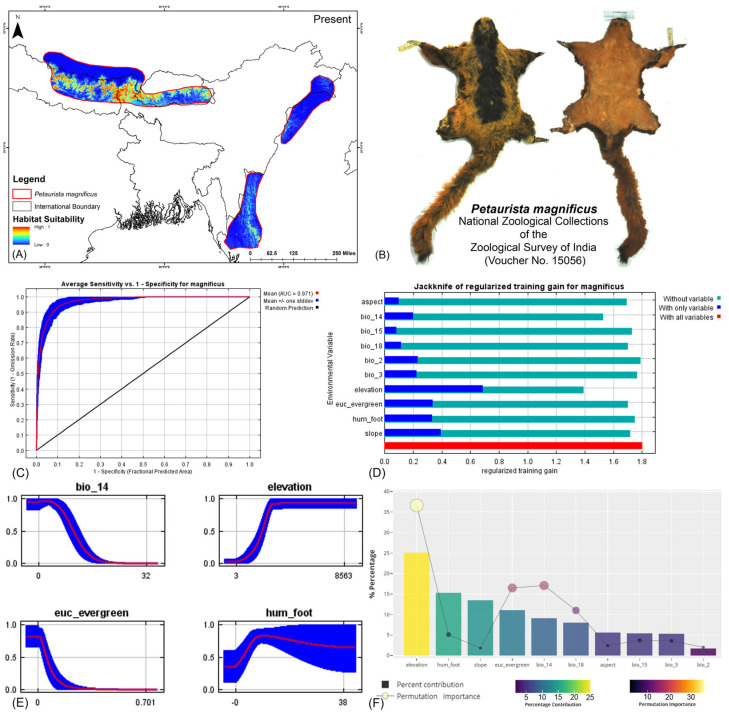
(**A**) Suitable area with IUCN extent delineated by the HCM approach for *Petaurista magnificus* in the present scenario. (**B**) Photo of the preserved stuffed skin of *P. magnificus* archived at the National Zoological Collections of the Zoological Survey of India. (**C**) Average training ROC (receiver operating characteristic) for the model. (**D**) Jackknife test for all the selected variables, where the blue bar shows the importance of each variable in explaining the data variation when used separately. The green bar shows the loss in overall gain after the particular variable was dropped. Red bar = total model gain. (**E**) Response curves of the major contributing predictors governing the habitat suitability of *P. magnificus*. (**F**) Percentage contribution and permutation importance of covariates.

**Figure 3:**
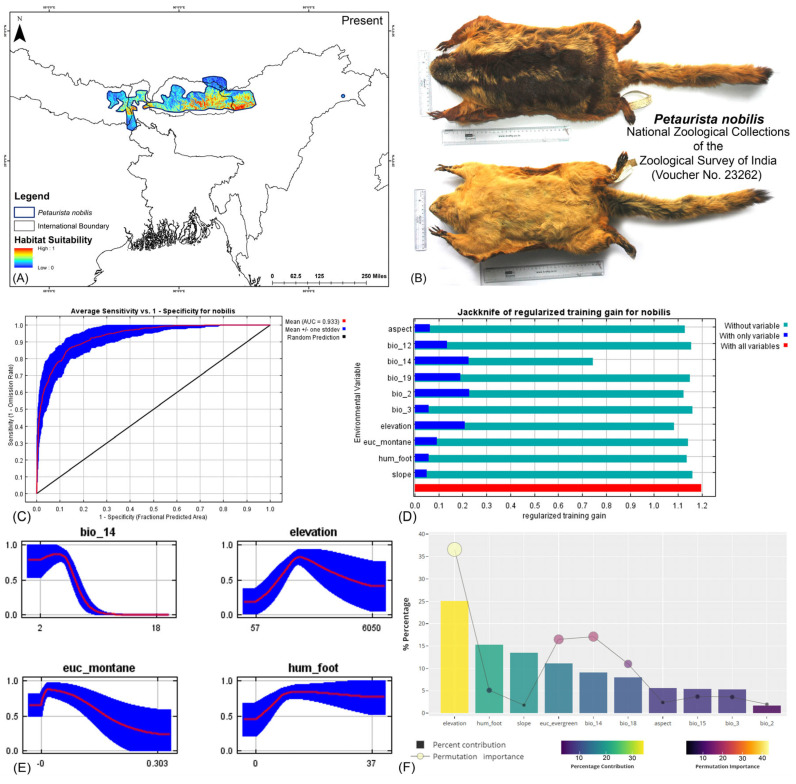
(**A**) Suitable area with IUCN extent delineated by the HCM approach for *Petaurista nobilis* in the present scenario. (**B**) Photo of the preserved stuffed skin of *P. nobilis* archived at the National Zoological Collections of the Zoological Survey of India. (**C**) Average training ROC (receiver operating characteristic) for the model. (**D**) Jackknife test for all the selected variables, where the blue bar shows the importance of each variable in explaining the data variation when used separately. The green bar shows the loss in overall gain after the particular variable was dropped. Red bar = total model gain. (**E**) Response curves of the major contributing predictors governing the habitat suitability of *P. nobilis*. (**F**) Percentage contribution and permutation importance of covariates.

Both modeling approaches demonstrated high predictive accuracy in terms of average training area under the curve (AUC) across multiple runs. Specifically for *P. magnificus*, the AUC values reached 0.971 ± 0.009 (HCM) and 0.968 ± 0.010 (COM), while for *P. nobilis*, the AUCs were 0.933 ± 0.021 (HCM) and 0.921 ± 0.030 (COM) (Figure 2, Figure 3, and Figures S3–S6). Given that both AUC values exceeded 0.9, the models exhibited exceptional performance in predicting species distributions. The habitat suitability analysis revealed that *P. magnificus* occupies approximately 7199 km^2^ (HCM) and 6909 km^2^ (COM) of suitable habitat, constituting only 3.92% (HCM) and 3.75% (COM) of its total IUCN-defined range. In comparison, *P. nobilis* was found to have 7998 km^2^ (HCM) and 5667 km^2^ (COM) of suitable habitat, representing 14.17% (HCM) and 10.04% (COM) of its IUCN range.

The model identified elevation as the most influential variable for *P. magnificus*, considering the highest 25.1% contribution and highest permutation importance of 36.6% with the the HCM approach (Table 1, Figures S6–S11). The Human Influence Index (hum_foot) emerged as a major contributing predictor, accounting for 15.3%; however, it exhibited relatively low permutation importance, accounting for only 5.1%, indicating a lesser impact on the final model’s predictive performance. The habitat variable, Euclidean distance to evergreen forests (euc_evergreen), contributed 11.1% with a permutation importance of 16.5%, followed by the bioclimatic variable precipitation of the driest month (bio_14), which contributed 9.1% (Table 1, Figures S5 and S6). Although the bioclimatic variable bio_4 had a low percentage contribution to the model, it demonstrated the second-highest permutation importance at 17.1%, highlighting its significant influence on the distribution. In the COM approach, elevation was identified as the most influential variable, contributing 35% to the model and exhibiting a permutation importance of 36%. Additionally, precipitation of the warmest quarter (bio_18) emerged as the second-most influential variable, with a contribution of 23.8% and a permutation importance of 23% in predicting the distribution of *P. magnificus* (Table 1, Figures S7, S10, and S11). In contrast, for *P. nobilis*, the bioclimatic variable precipitation of the driest month (bio_14) was the most significant predictor, contributing 34.3% and peak permutation importance of 43.4%. Moreover, elevation contributed 9.4% with an importance of 12.5%. The habitat variable Euclidean distance to montane forests (euc_montane) accounted for 4% of the contribution with a 2.5% permutation importance. Additionally, the anthropogenic variable Human Influence Index (hum_foot) contributed 5% with a permutation importance of 4% (Table 1, Figures S5 and S6). Similarly, the COM approach yielded consistent findings, with precipitation of the driest month (bio_14) emerging as the most influential variable for the distribution of *P. nobilis*, exhibiting the highest contribution of 36% and the highest permutation importance of 42.9%. Although elevation had a relatively lower contribution of 12.4% compared to other bioclimatic variables such as mean diurnal range (bio_2) and annual precipitation (bio_12), it ranked as the second-most important variable in terms of permutation importance, with a value of 23.8% (Table 1, Figures S7, S10, and S11). These findings highlight the varying environmental and anthropogenic factors influencing the distribution of both species, underscoring the need for species-specific conservation strategies.

**Figure 4:**
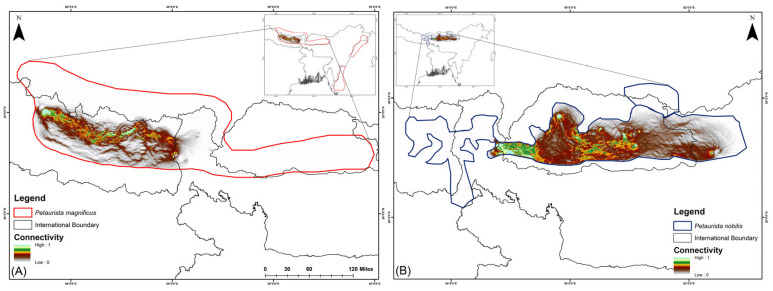
Biological corridor connectivity simulation for two flying squirrel species within their IUCN extent under the present scenario in the HCM approach. (**A**) *Petaurista magnificus*, (**B**) *Petaurista nobilis*.

In the current scenario, habitat connectivity for *P. magnificus* is primarily restricted to eastern Nepal and the Nepal–India border region, with a mean connectivity value of 0.1118 (Figure 4). In contrast, for *P. nobilis*, corridor connectivity was identified within Bhutan and across transboundary regions, specifically between Bhutan and Arunachal Pradesh (India) as well as Bhutan and Sikkim (India), with a mean connectivity value of 0.6368 (Figure 4).

### 3.2. Future Habitat Dynamics and Centroid Shift in Climate Change Scenarios

**Figure 5:**
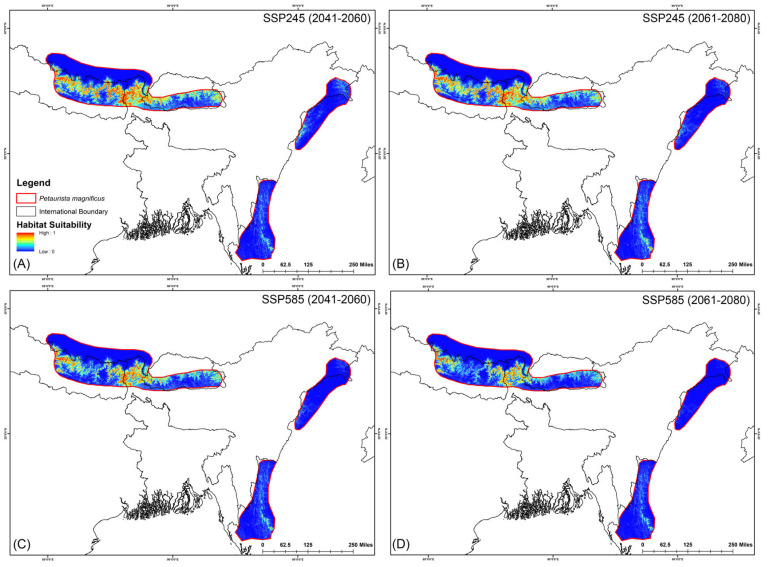
Suitable habitats for *Petaurista magnificus* within the IUCN extent under different future climatic scenarios with HCM approach. (**A**,**B**) represent SSP245, and (**C**,**D**) represent SSP585 for 2041–2060 and 2061–2080, respectively.

**Figure 6:**
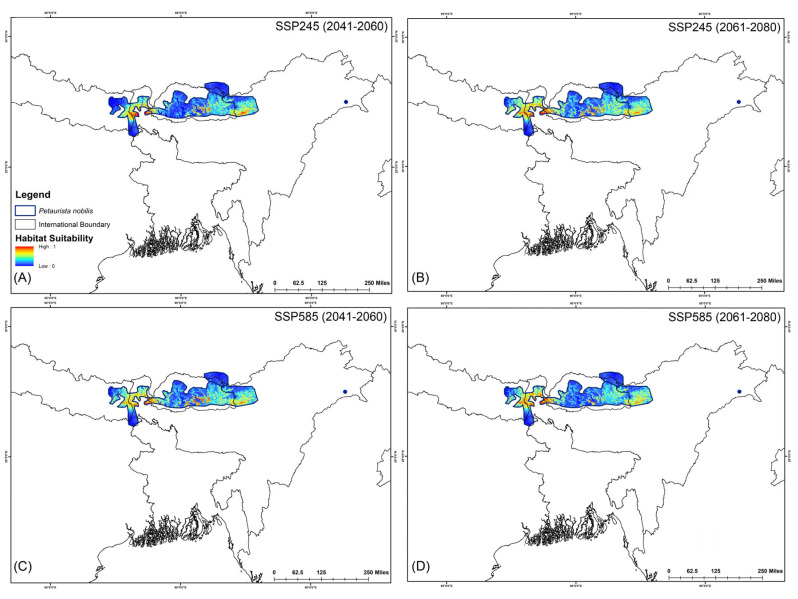
Suitable habitats for *Petaurista nobilis* within the IUCN extent under different future climatic scenarios with HCM approach. (**A**,**B**) represent SSP245, and (**C**,**D**) represent SSP585 for 2041–2060 and 2061–2080, respectively.

The assessment of habitat dynamics under future climatic scenarios reveals alarming trends, with a substantial loss of suitable habitats of up to 89.88% due to climate change. Notably, both SSP scenarios indicate the most severe habitat decline occurring during the 2061–2080 period for both species (Table 2, Figure 5 and Figure 6). Specifically, for *P. magnificus*, the projected reduction in suitable habitat ranges from 9.43% to 81.90%, while for *P. nobilis*, the decline is even more pronounced, ranging from 85.97% to 89.88% (Table 2, Figure 5 and Figure 6). Under SSP245 (2041–2060), habitat loss is estimated at 9.43% for *P. magnificus* and 85.97% for *P. nobilis*. Moreover, in the SSP245 (2061–2080) scenario, the decline increases to 18.73% and 86.37%, respectively. Furthermore, the most severe losses are projected under the SSP585 scenario, where habitat decline reaches 58.14% and 89.49% for *P. magnificus* and *P. nobilis*, respectively, during 2041–2060, escalating further to 81.90% and 89.88% by 2061–2080 (Table 2, Figure 5 and Figure 6).

**Figure 7:**
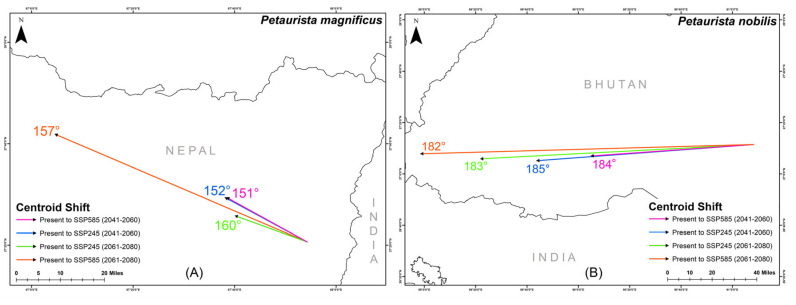
Centroid shift for two flying squirrel species from present to different future climatic scenarios within their IUCN extent with HCM approach. (**A**) *Petaurista magnificus*, (**B**) *Petaurista nobilis*.

The centroid of suitable habitat for *P. magnificus* is currently located in eastern Nepal, while for *P. nobilis*, it is situated in central Bhutan (Figure 7). However, projections indicate a significant centroid shift for both species in response to future climate change. Specifically, *P. magnificus* is expected to experience a northwestward shift, with directional changes ranging between 151° and 160° across different future climatic scenarios (Figure 7). Similarly, *P. nobilis* is projected to shift westward, with centroid movement ranging between 182° and 185° in the future (Figure 7).

### 3.3. Habitat Quality and Shape Geometry

The assessment of habitat patch geometry for both species indicates severe fragmentation and habitat loss due to climate change (Table 3). Specifically, for *P. magnificus*, a significant reduction in suitable patches is observed, with the NPs decreasing by up to 43.62% and PD declining by 35.34%. Additionally, patch sizes shrink considerably, as evidenced by a 10% to 58% reduction in the LPI, while the LSI decreases by over 6.3%, indicating a shift towards simpler geometric shapes. Furthermore, habitat proximity increases, as reflected in a 30.80% reduction in the AI, which suggests increased isolation of remaining patches (Table 3). Similarly, *P. nobilis* experiences pronounced habitat fragmentation, with a reduction of over 51% in suitable patches under future climatic scenarios. The patch density declines drastically, by as much as 93.19%, and the remaining patches become smaller and more geometrically simplified, as indicated by reductions of over 8.4% in LPI and 42.94% in LSI (Table 3). Additionally, these patches become more dispersed, with AI decreasing between 19.03% and 42.08%. Collectively, these findings highlight the severe impact of climate change on habitat structure for both species, leading to the loss of viable patches and the formation of smaller, simpler, and more isolated habitat fragments (Table 3).

## 4. Discussion

Given the ongoing global decline in biodiversity, mammal populations are increasingly impacted by both environmental changes and direct and indirect anthropogenic pressures [81]. Moreover, the transboundary regions are particularly sensitive to these human-induced disturbances, necessitating collaborative conservation efforts among neighboring nations [82]. Therefore, the implementation of a well-coordinated transboundary conservation management plan, involving multiple stakeholders working in unison, is critical for ensuring effective biodiversity protection. In this context, identifying suitable habitats for flying squirrel species—considering bioclimatic, ecological, and anthropogenic factors—is essential for guiding transboundary conservation strategies [83]. Such research contributes to the development of joint conservation initiatives, supporting ongoing collaborative efforts such as the India–Nepal Terai Arc Landscape (TAL) and the India–Bhutan Manas Tiger Reserve, which are actively engaged in transboundary wildlife conservation [84].

The IUCN SSC Small Mammal Specialist Group (SMSG) has delineated the range boundaries for both *P. magnificus* and *P. nobilis* based on confirmed presence records [30,31]. However, the present assessment found that only 3.92% of the IUCN-designated range is suitable for *P. magnificus* and 14.17% for *P. nobilis*, highlighting a significant disparity in habitat suitability within their estimated extent. This discrepancy is concerning and underscores the need for more targeted and focused conservation efforts. Furthermore, the forests of South and Southeast Asia are undergoing rapid deforestation exacerbated by climate change, posing a severe threat to both species [85]. This habitat loss is particularly alarming. Given their strong dependence on forested habitats, evergreen forests contribute 11.1% to the habitat suitability of *P. magnificus*, while montane forests contribute 4% for *P. nobilis*. Additionally, both species exhibit a negative relation with increasing distance from forested areas, indicating that their distribution declines as they move farther from suitable forest habitats. This reinforces the conclusion that these species are highly reliant on the transboundary forested landscapes [31,32]. Among climatic factors, precipitation of the driest month (bio_14) plays a crucial role in determining habitat suitability, contributing 9.1% for *P. magnificus* and 34.3% for *P. nobilis*. These findings align with previous studies emphasizing the significance of bioclimatic variables of other flying squirrel species, including *P. philippensis, P. mishmiensis*, and *P. mechukaensis* [44,45,86,87,88]. Additionally, elevation is a key factor, contributing 25.1% to *P. magnificus*, which is higher than its 9.4% contribution to *P. nobilis*, indicating that *P. magnificus* occupies higher elevations than *P. nobilis*. Moreover, the anthropogenic variable Human Influence Index also influences habitat suitability, as it had a greater impact on *P. magnificus* than on *P. nobilis*. This may be attributed to the broader distribution range of *P. magnificus*, which brings it into closer proximity to human settlements.

Furthermore, the identified transboundary corridors for both species require on-ground assessments, as limited connectivity may hinder their movement. The reduced connectivity in these corridors can be attributed to geographical barriers such as riverine systems present in that rugged landscape [89,90]. While these flying squirrels possess the ability to glide, wide river spans may still act as significant obstacles to movement. As a result, restricted dispersal may lead to increased intraspecific and interspecific competition, reduced genetic diversity, and a higher risk of inbreeding. Compounding this issue, climate change has further exacerbated habitat loss, significantly reducing the availability of suitable areas within their range. Furthermore, the projections indicate that habitat suitability for *P. magnificus* is expected to decline by 9.43% to 81.90%, while *P. nobilis* faces an even greater reduction of 85.97% to 89.88% under future climate scenarios. This consistent trend of habitat loss driven by climate change and aligns with patterns seen in other sympatric species within the same genus [45]. Moreover, these findings align with previous studies demonstrating that high-elevation species are particularly vulnerable to climate shifts, leading to drastic declines in suitable habitat [91,92,93]. The loss of suitable areas is especially concerning given that both species already inhabit relatively small and isolated ranges. This corroborates prior research indicating that species with restricted distributions are more susceptible to climate-induced habitat loss, further increasing their extinction risk [94,95,96]. In addition to habitat reduction, climate change has intensified habitat fragmentation across the species’ ranges. Many viable habitat patches are projected to be entirely lost, while those that persist are expected to be smaller and more spatially isolated. These fragmented patches are critical for the survival of these endemic flying squirrels, emphasizing the urgent need for targeted conservation efforts. Given the already low connectivity and the increasing fragmentation of suitable habitats, the vulnerability of these species is significantly heightened. Conservation strategies must prioritize maintaining and restoring connectivity between habitat patches to prevent further population declines and ensure their long-term survival [97,98].

## 5. Limitations and Recommendations

This study assessed habitat suitability for the two *Petaurista* species using two modeling approaches, viz. HCM and COM. Furthermore, given the holistic efficacy and relevance in integrating both ecological and environmental variables, habitat connectivity, future climate change vulnerability, habitat quality, shape geometry, and centroid shift were analyzed based on the HCM approach. However, certain limitations must be acknowledged, as the species’ sporadic and limited records pose challenges for model validation, and the probabilistic nature of SDM means that slight variations in input parameters could have influenced the outcomes. Despite these constraints, this study establishes a strong foundation for further research and conservation efforts. When integrated with extensive field validation, these findings will contribute significantly to the protection and long-term survival of these little-known species. Considering the vulnerability of the two *Petaurista* species to climate change and other threats within their range, as identified in the present study, several measures should be implemented for their effective conservation and management. Hence, rigorous field surveys are essential to confirm their presence both within and beyond their range, thereby providing deeper insights into their ecology. Such assessments will strengthen species distribution and ecological modeling efforts, thereby enhancing conservation management strategies. In addition to field surveys, acquiring genetic data is crucial for understanding population structure, gene flow, and phylogenetic relationships. Furthermore, corridor connectivity assessments should also be conducted to evaluate the degree of habitat fragmentation and the isolation of viable populations. Such field expeditions should prioritize areas identified as suitable in the present study, assessing habitat viability and investigating the presence of other sympatric species. Given the transboundary distribution of these flying squirrels, collaborative conservation initiatives must be established, extending to other sympatric species inhabiting the same landscapes. Such effective conservation efforts require active community involvement in collaboration with conservation organizations such as the IUCN SSC SMSG, national forest departments, and other governmental agencies. Moreover, regular transboundary dialogues should be held to monitor and protect forest habitats, ensuring coordinated conservation actions. Furthermore, national forest departments should receive support from governmental and non-governmental organizations, research institutions, and conservation networks to facilitate comprehensive assessments and implement targeted conservation strategies. Additionally, environmental impact assessments (EIAs) must be conducted for any developmental projects within these ecologically fragile landscapes to mitigate potential threats. A community-based approach is also critical, involving training programs and awareness campaigns to educate local populations on nature and wildlife conservation. Hence, to reinforce conservation efforts, the formation of transboundary joint committees with the responsibility of coordinating protection measures across these landscapes is recommended. These committees should include village leaders, forest personnel, defense personnel, naturalists, scientists, and other key stakeholders, ensuring a holistic and community-driven approach to conservation. Their role would be pivotal in monitoring wildlife, facilitating conservation activities, and fostering community participation. Ultimately, safeguarding both current and future suitable habitat patches is imperative to mitigate extinction risks. Therefore, by addressing these priorities, the present study provides valuable guidance for future field surveys and contributes to the formulation of robust species management plans for the conservation of these unique flying squirrels.

## 6. Conclusions

The Eastern Himalayas are a biodiversity hotspot home to numerous endemic species. This study employed SDM to delineate the suitable habitats of two understudied flying squirrel species utilizing two modeling approaches (HCM and COM). Furthermore, the present connectivity in their habitats, along with future dynamics of suitable habitats, habitat quality, shape geometry, and centroid shifts under future scenarios, were assessed using the HCM approach. The HCM approach was specifically selected for its holistic representation, as it integrates both ecological and environmental parameters relevant to the species. The findings reveal alarming trends, with both species experiencing a significant decline in suitable habitat due to climate change. The key climatic parameters influencing their distribution were identified, also underscoring their strong dependence on forested ecosystems. Based on these findings, several recommendations are proposed, including genetic assessments to understand population structure and gene flow, corridor connectivity evaluations to assess habitat fragmentation, and rigorous field studies to validate model predictions. Additionally, the establishment of joint forest conservation committees—comprising local communities, forest personnel, defense personnel, naturalists, and scientists—is strongly encouraged. Enhanced institutional support for forest departments from governmental agencies, NGOs, and conservation organizations is also emphasized to facilitate effective conservation strategies. This study provides crucial insights for guiding future field research across the transboundary landscapes of the Eastern Himalayas. It serves as a valuable resource for the development of comprehensive species management plans aimed at safeguarding these elusive flying squirrel species.

## Figures and Tables

**Table 1:** Variable importance and permutation importance for the two flying squirrels in the two modeling approaches: habitat–climate model (HCM) and climate-only model (COM). Mean diurnal range (mean of monthly (max temp–min temp)): bio_2; isothermality (BIO2/BIO7) (×100): bio_3; annual precipitation: bio_12; precipitation of driest month: bio_14; precipitation seasonality (coefficient of variation): bio_15; precipitation of warmest quarter: bio_18; precipitation of coldest quarter: bio_19; Euclidean distance to evergreen forests: euc_evergreen; Euclidean distance to montane forests: euc_montane; Human Influence Index: hum_foot.

Species	Modeling Approach	Variables	Percentage Contribution	Permutation Importance
*Petaurista magnificus*	HCM	elevation	25.1	36.6
		hum_foot	15.3	5.1
		slope	13.5	1.8
		euc_evergreen	11.1	16.5
		bio_14	9.1	17.1
		bio_18	8	11
		aspect	5.6	2.4
		bio_15	5.4	3.7
		bio_3	5.3	3.6
		bio_2	1.7	2
	COM	elevation	35.7	36
		bio_18	23.8	23.12
		bio_14	11.6	17
		bio_3	8.4	5
		bio_15	7.6	7.1
		bio_2	6.5	6.2
		aspect	6.4	5.5
*Petaurista nobilis*	HCM	bio_14	34.3	43.4
		bio_2	16.1	12.9
		bio_19	10.3	10.1
		elevation	9.4	12.5
		bio_12	8	4.7
		aspect	6.1	4.4
		hum_foot	5	4
		euc_montane	4	2.5
		bio_3	3.7	3.9
		slope	3	1.6
	COM	bio_14	36	42.9
		bio_2	18.2	9.1
		bio_12	15.7	11.2
		elevation	12.4	23.8
		bio_19	7.4	4.5
		aspect	5.8	2.8
		bio_3	4.5	5.8

**Table 2:** Suitable area (in km^2^) within the IUCN extent of two flying squirrels in present and future climatic scenarios based on HCM approach.

Scenario	*Petaurista magnificus*	*Petaurista nobilis*
SSP245 (2041–2060)	6520	1122
SSP245 (2061–2080)	5850	1090
SSP585 (2041–2060)	3013	840
SSP585 (2061–2080)	1303	809

**Table 3:** Assessment of habitat shape geometry of two flying squirrels in present and future scenarios with HCM approach. NPs: number of patches, PD: patch density, LPI: largest patch index, LSI: landscape shape index, AI: aggregation index.

Species	Scenario	NPs	PD	LPI	LSI	AI
*Petaurista magnificus*	Present	392	850,812,612.9	31.5322	29.6824	65.7295
	SSP245 (2041–2060)	378	785,405,585.1	28.3777	27.8103	63.9378
	SSP245 (2061–2080)	359	658,867,521.4	25.9658	25.9869	61.5918
	SSP585 (2041–2060)	316	638,732,160.6	11.1849	25.4091	54.6146
	SSP585 (2061–2080)	221	550,134,305.4	13.1236	19.9178	45.4797
*Petaurista nobilis*	Present	285	556,779,820	25.3563	25.2458	72.5612
	SSP245 (2041–2060)	139	355,717,468.8	23.2228	14.403	58.7506
	SSP245 (2061–2080)	125	181,995,412.8	17.4312	11.1045	48.9352
	SSP585 (2041–2060)	130	92,410,714.29	14.0476	16.5517	44.3896
	SSP585 (2061–2080)	118	37,886,279.36	16.1928	10.8772	42.0243

## Data Availability

The data used in the analysis can be provided upon request to the corresponding authors.

## Supplementary Materials

The following supporting information can be downloaded at https://www.mdpi.com/article/10.3390/life15040589/s1. Figure S1. Correlations between the covariates chosen for the habitat–climate model for *P. magnificus*. The Pearson correlation coefficient is primarily used here. However, where the Spearman or Kendall correlation coefficient exceeds the Pearson correlation coefficient, an “s” or “k” is displayed in the bottom-right corner of the variable box. Figure S2. Correlations between the covariates chosen for the habitat–climate model for *P. nobilis*. The Pearson correlation coefficient is primarily used here. However, where the Spearman or Kendall correlation coefficient exceeds the Pearson correlation coefficient, an “s” or “k” is displayed in the bottom-right corner of the variable box. Figure S3. Correlations between the covariates chosen for the climate-only model for *P. magnificus*. The Pearson correlation coefficient is primarily used here. However, where the Spearman or Kendall correlation coefficient exceeds the Pearson correlation coefficient, an “s” or “k” is displayed in the bottom-right corner of the variable box. Figure S4. Correlations between the covariates chosen for the climate-only model for *P. nobilis*. The Pearson correlation coefficient is primarily used here. However, where the Spearman or Kendall correlation coefficient exceeds the Pearson correlation coefficient, an “s” or “k” is displayed in the bottom-right corner of the variable box. Figure S5. Climate-only modeling approach for the two *Petaurista* species. (A) Habitat suitability maps and (B) receiver operating characteristic (ROC) curves and area under the curve (AUC) evaluation plot for *P. magnificus*. (C) Habitat suitability maps and (D) receiver operating characteristic (ROC) curves and area under the curve (AUC) evaluation plot for *P. nobilis*. Figure S6. Curves showing the training omission rate and predicted area as a function of the cumulative threshold, averaged over the replicate runs for the two *Petaurista* species under two different modeling approaches. Figure S7. Jackknife test for all the selected variables in the climate-only model, where the blue bar shows the importance of each variable in explaining the data variation where used separately. The green bar shows the loss in overall gain after the particular variable was dropped. Red bar = total model gain. (A) *P. magnificus* and (B) *P. nobilis*. Figure S8. Curves showing how each environmental variable affects the Maxent prediction for *P. magnificus* in the habitat–climate model; how the predicted probability of presence changes as each environmental variable is varied, keeping all other environmental variables at their average sample value; the marginal effect of changing exactly one variable, whereas the model may take advantage of sets of variables changing together; and the mean response of the 50 replicate Maxent runs (red) and the mean +/− one standard deviation (blue, two shades for categorical variables). Figure S9. Curves showing how each environmental variable affects the Maxent prediction for *P. nobilis* in the habitat–climate model; how the predicted probability of presence changes as each environmental variable is varied, keeping all other environmental variables at their average sample value; the marginal effect of changing exactly one variable, whereas the model may take advantage of sets of variables changing together; and the mean response of the 50 replicate Maxent runs (red) and the mean +/− one standard deviation (blue, two shades for categorical variables). Figure S10. Curves showing how each environmental variable affects the Maxent prediction for *P. magnificus* in the climate-only model; how the predicted probability of presence changes as each environmental variable is varied, keeping all other environmental variables at their average sample value; the marginal effect of changing exactly one variable, whereas the model may take advantage of sets of variables changing together; and the mean response of the 50 replicate Maxent runs (red) and the mean +/− one standard deviation (blue, two shades for categorical variables). Figure S11. Curves showing how each environmental variable affects the Maxent prediction for *P. nobilis* in the climate-only model; how the predicted probability of presence changes as each environmental variable is varied, keeping all other environmental variables at their average sample value; the marginal effect of changing exactly one variable, whereas the model may take advantage of sets of variables changing together; and the mean response of the 50 replicate Maxent runs (red) and the mean +/− one standard deviation (blue, two shades for categorical variables). Table S1. Locality information on *P. magnificus* and *P. nobilis* specimens archived at the National Zoological Collections of the Zoological Survey of India, Kolkata, India.

## References

[1] Ripple W.J., Newsome T.M., Wolf C., Dirzo R., Everatt K.T., Galetti M., Hayward M.W., Kerley G.I.H., Levi T., Lindsey P.A. (2015). Collapse of the world’s largest herbivores. Sci. Adv..

[2] Abedin I., Mukherjee T., Kim A.R., Lee S.R., Kim H.-W., Kundu S. (2024). Fragile futures: Evaluating habitat and climate change response of hog badgers (Mustelidae: *Arctonyx*) in the conservation landscape of mainland Asia. Ecol. Evol..

[3] Santangeli A., Mammola S., Lehikoinen A., Rajasärkkä A., Lindén A., Saastamoinen M. (2022). The effects of protected areas on the ecological niches of birds and mammals. Sci. Rep..

[4] Jha S., Bawa K.S. (2006). Population growth, human development, and deforestation in biodiversity hotspots. Conserv. Biol..

[5] Kiene F., Andriatsitohaina B., Ramsay M.S., Rakotondravony R., Strube C., Radespiel U. (2021). Habitat fragmentation and vegetation structure impact gastrointestinal parasites of small mammalian hosts in Madagascar. Ecol. Evol..

[6] Ceballos G., Ehrlich P.R., Barnosky A.D., García A., Pringle R.M., Palmer T.M. (2015). Accelerated modern human-induced species losses: Entering the sixth mass extinction. Sci. Adv..

[7] Conde D.A., Staerk J., Colchero F., da Silva R., Schöley J., Baden H.M., Jouvet L., Fa J.E., Syed H., Jongejans E. (2019). Data gaps and opportunities for comparative and conservation biology. Proc. Natl. Acad. Sci. USA.

[8] Mokany K., Ferrier S., Harwood T.D., Ware C., Di Marco M., Grantham H.S., Venter O., Hoskins A.J., Watson J.E.M. (2020). Reconciling global priorities for conserving biodiversity habitat. Proc. Natl. Acad. Sci. USA.

[9] Meehl G.A., Covey C., Delworth T., Latif M., McAvaney B., Mitchell J.F.B., Stouffer R.J., Taylor K.E. (2007). The WCRP CMIP3 Multimodel Dataset: A New Era in Climate Change Research. Bull. Am. Meteorol. Soc..

[10] Belden G., Stuebing R., Nyegang M. (2007). Small carnivores in mixed-use forest in Bintulu Division, Sarawak, Malaysia. Small Carniv. Conserv..

[11] Datta A., Naniwadekar R., Anand M.O. (2008). Occurrence and conservation status of small carnivores in two protected areas in Arunachal Pradesh, north-east India. Small Carniv. Conserv..

[12] Chutipong W., Lynam A.J., Steinmetz R., Savini T., Gale G.A. (2014). Sampling mammalian carnivores in western Thailand: Issues of rarity and detectability. Raffles Bull. Zool..

[13] Abedin I., Mukherjee T., Kim A.R., Kim H.-W., Lee S.R., Kundu S. (2025). Shifting shadows: Assessing the habitat and climate change response of binturong (*Arctictis binturong*) in the conservation landscape of the Asian continent. Ecol. Inform..

[14] Ahmad S., Yang L., Khan T.U., Wanghe K., Li M., Luan X. (2020). Using an ensemble modelling approach to predict the potential distribution of Himalayan gray goral (*Naemorhedus goral bedfordi*) in Pakistan. Glob. Ecol. Conserv..

[15] Koli V.K. (2016). Biology and conservation status of flying squirrels (*Pteromyini*, Sciuridae, Rodentia) in India: An update and review. Proc. Zool. Soc..

[16] Carey A.B., Harrington C.A. (2001). Small mammals in young forests: Implications for management for sustainability. For. Ecol. Manag..

[17] Nandini R., Parthasarathy N. (2008). Food habits of the Indian giant flying squirrel (*Petaurista philippensis*) in a rain forest fragment, Western Ghats. J. Mammal..

[18] Koprowski J.L., Goldstein E.A., Bennet K.R., Pereira Mendes C., Wilson D.E., Lacher T.E., Mittermeier R.A. (2016). Family Sciuridae (tree, flying and ground squirrels, chipmunks, marmots, and prairie dogs). Handbook of the Mammals of the World. 6. Lagomorphs and Rodents I.

[19] Casanovas-Vilar I., Garcia-Porta J., Fortuny J., Sanisidro Ó., Prieto J., Querejeta M., Llácer S., Robles J.M., Bernardini F., Alba D.M. (2018). Oldest skeleton of a fossil flying squirrel casts new light on the phylogeny of the group. eLife.

[20] Burgin C.J., Colella J.P., Kahn P.L., Upham N.S. (2018). How many species of mammals are there?. J. Mammal..

[21] Dudley R., Byrnes G., Yanoviak S.P., Borrell B., Brown R.M., McGuire J.A. (2007). Gliding and the functional origins of flight: Biomechanical novelty or necessity?. Annu. Rev. Ecol. Evol. Syst..

[22] Chaitanya R., McGuire J.A., Karanth P., Meiri S. (2023). Their fates intertwined: Diversification patterns of the Asian gliding vertebrates may have been forged by dipterocarp trees. Proc. R. Soc. B.

[23] Byrnes G., Spence A.J. (2011). Ecological and biomechanical insights into the evolution of gliding in mammals. Integr. Comp. Biol..

[24] McGuire J.A., Dudley R. (2011). The biology of gliding in flying lizards (genus *Draco*) and their fossil and extant analogs. Integr. Comp. Biol..

[25] Lin Y.S., Progulske D.R., Lee P.F., Day Y.T. (1985). Bibliography of *Petauristinae* (Rodentia, Sciuridae). J. Taiwan Mus..

[26] Lee P.F., Liao C.Y. (1998). Species richness and research trend of flying squirrels. J. Taiwan Mus..

[27] Umapathy G., Kumar A. (2000). The occurrence of arboreal mammals in the rain forest fragments in Anamalai Hills, south India. Biol. Conserv..

[28] Kumara H.N., Singh M. (2006). Distribution and relative abundance of giant squirrels and flying squirrels in Karnataka, India. Mammalia.

[29] Puyravaud J.P., Davidar P., Laurance W.F. (2010). Cryptic destruction of India’s native forests. Conserv. Lett..

[30] Sharma G., Kamalakannan M., Saikia U., Talmale S., Dam D., Banerjee D. (2024). Checklist of Fauna of India: Chordata: Mammalia. Version 1.0. Zoological Survey of Indial.

[31] Molur S. (2016). Petaurista magnificus. The IUCN Red List of Threatened Species.

[32] Molur S. (2016). Petaurista nobilis. The IUCN Red List of Threatened Species.

[33] Johnsingh A.J.T., Manjrekar N. (2015). Mammals of South Asia, Vol. 2.

[34] Menon V. (2014). Indian Mammals—A Field Guide.

[35] Ellerman J.R., Morrison-Scot T.C.S. (1966). Checklist of Palaearctic and Indian Mammals 1758 to 1956.

[36] Ortega-Huerta M.A., Peterson A.T. (2004). Modelling spatial patterns of biodiversity for conservation prioritization in North-Eastern Mexico. Divers. Distrib..

[37] Guisan A., Zimmermann N.E. (2000). Predictive habitat distribution models in ecology. Ecol. Model..

[38] Elith J., Leathwick J.R. (2009). Species distribution models: Ecological explanation and prediction across space and time. Annu. Rev. Ecol. Evol. Syst..

[39] Pearson R.G. (2010). Species’ distribution modeling for conservation educators and practitioners. Netw. Conserv. Educ. Pract. Cent. Biodivers. Conserv. Am. Mus. Nat. Hist..

[40] Kujala H., Moilanen A., Araújo M.B., Cabeza M. (2013). Conservation planning with uncertain climate change projections. PLoS ONE.

[41] Eyre A.C., Briscoe N.J., Harley D.K.P., Lumsden L.F., McComb L.B., Lentini P.E. (2022). Using species distribution models and decision tools to direct surveys and identify potential translocation sites for a critically endangered species. Divers. Distrib..

[42] Hu W., Onditi K.O., Jiang X., Wu H., Chen Z. (2022). Modeling the potential distribution of two species of shrews (*Chodsigoa hypsibia* and *Anourosorex squamipes*) under climate change in China. Diversity.

[43] Abedin I., Mukherjee T., Singha H., Go Y., Kang H.-E., Kim H.-W., Kundu S. (2025). Predicting climate-driven habitat dynamics of adjutants for implementing strategic conservation measures in South and Southeast Asian landscapes. Sci. Rep..

[44] Koli V.K., Jangid A.K., Singh C.P. (2023). Habitat suitability mapping of the Indian giant flying squirrel (*Petaurista philippensis* Elliot, 1839) in India with ensemble modeling. Acta Ecol. Sin..

[45] Abedin I., Kamalakannan M., Mukherjee T., Choudhury A., Singha H., Abedin J., Banerjee D., Kim H.-W., Kundu S. (2025). Fading into Obscurity: Impact of Climate Change on Suitable Habitats for Two Lesser-Known Giant Flying Squirrels (Sciuridae: *Petaurista*) in Northeastern India. Biology.

[46] Hosseini N., Ghorbanpour M., Mostafavi H. (2024). The influence of climate change on the future distribution of two *Thymus* species in Iran: MaxEnt model-based prediction. BMC Plant Biol..

[47] Sanguet A., Wyler N., Petitpierre B., Honeck E., Poussin C., Martin P., Lehmann A. (2022). Beyond topo-climatic predictors: Does habitats distribution and remote sensing information improve predictions of species distribution models?. Glob. Ecol. Conserv..

[48] Molur S., Srinivasulu C., Srinivasulu B., Walker S., Nameer P.O., Ravikumar L. (2005). Status of Non-Volant Small Mammals: Conservation Assessment and Management Plan (C.A.M.P) Workshop Report.

[49] Smith A.T., Xie Y. (2008). A Guide to the Mammals of China.

[50] Thorington R.W., Hoffmann R.S., Wilson D.E., Reader D.M. (2005). Family Sciuridae. Mammal Species of the World.

[51] Choudhury A.U. (2002). *Petaurista nobilis singhei*—First record in India and a note on its taxonomy. J. Bombay Nat. Hist. Soc..

[52] Choudhury A.U. (2009). Five possible additions to the mammals of China. Newslett. J. Rhino Found. Nat. NE India.

[53] Bachman S., Moat J., Hill A.W., de la Torre J., Scott B. (2011). Supporting Red List threat assessments with GeoCAT: Geospatial conservation assessment tool. ZooKeys.

[54] Brown J.L., Bennett J.R., French C.M. (2017). SDMtoolbox 2.0: The next generation Python-based GIS toolkit for landscape genetic, biogeographic, and species distribution model analyses. PeerJ.

[55] Critical Ecosystem Partnership Fund. https://www.cepf.net/.

[56] Peterson A.T., Soberón J. (2012). Species distribution modeling and ecological niche modeling: Getting the concepts right. Braz. J. Nat. Conserv..

[57] Fick S.E., Hijmans R.J. (2017). WorldClim 2: New 1-km spatial resolution climate surfaces for global land areas. Int. J. Climatol..

[58] Karra K., Kontgis C., Statman-Weil Z., Mazzariello J.C., Mathis M., Brumby S.P. (2021). Global Land Use/Land Cover with Sentinel-2 and Deep Learning. Proceedings of the IGARSS 2021—2021 IEEE International Geoscience and Remote Sensing Symposium.

[59] SEDAC. Last of the Wild Project, Version 2, 2005 (LWP-2): Global Human Footprint Dataset (Geographic). https://catalog.data.gov/dataset/last-of-the-wild-project-version-2-2005-lwp-2-last-of-the-wild-dataset-geographic.

[60] Morisette J.T., Jarnevich C.S., Holcombe T.R., Talbert C.B., Ignizio D., Talbert M.K., Silva C., Koop D., Swanson A., Young N.E. (2013). VisTrails SAHM: Visualization and workflow management for species habitat modeling. Ecography.

[61] Warren D.L., Glor R.E., Turelli M. (2010). ENMTools: A toolbox for comparative studies of environmental niche models. Ecography.

[62] Andrews M.B., Ridley J.K., Wood R.A., Andrews T., Blockley E.W., Booth B., Burke E., Dittus A.J., Florek P., Gray L.J. (2020). Historical simulations with HadGEM3-GC3.1 for CMIP6. J. Adv. Model. Earth Syst..

[63] Li L., Xie F., Yuan N. (2023). On the long-term memory characteristic in land surface air temperatures: How well do CMIP6 models perform?. Atmos. Ocean. Sci. Lett..

[64] Gautam S., Shany V.J. (2024). Navigating climate change in southern India: A study on dynamic dry-wet patterns and urgent policy interventions. Geosyst. Geoenviron..

[65] Allen B.J., Hill D.J., Burke A.M., Clark M., Marchant R., Stringer L.C., Williams D.R., Lyon C. (2024). Projected future climatic forcing on the global distribution of vegetation types. Philos. Trans. R. Soc. B Biol. Sci..

[66] Abedin I., Mukherjee T., Kim A.R., Kim H.-W., Kang H.-E., Kundu S. (2024). Distribution model reveals rapid decline in habitat extent for endangered hispid hare: Implications for wildlife management and conservation planning in future climate change scenarios. Biology.

[67] Breiner F.T., Nobis M.P., Bergamini A., Guisan A. (2018). Optimizing ensembles of small models for predicting the distribution of species with few occurrences. Methods Ecol. Evol..

[68] Elith J., Kearney M., Phillips S. (2010). The art of modelling range-shifting species. Methods Ecol. Evol..

[69] Abedin I., Mukherjee T., Kang H.E., Yoon T.H., Kim H.W., Kundu S. (2024). Unraveling the unknown: Adaptive spatial planning to enhance climate resilience for the endangered Swamp Grass-babbler (*Laticilla cinerascens*) with habitat connectivity and complexity approach. Heliyon.

[70] Kundu S., Mukherjee T., Kamalakannan M., Barhadiya G., Ghosh C., Kim H. (2023). Matrilineal phylogeny and habitat suitability of the endangered spotted pond turtle (*Geoclemys hamiltonii*; Testudines: Geoemydidae): A two-dimensional approach to forecasting future conservation consequences. PeerJ.

[71] Meller L., Cabeza M., Pironon S., Barbet-Massin M., Maiorano L., Georges D., Thuiller W. (2014). Ensemble distribution models in conservation prioritization: From consensus predictions to consensus reserve networks. Divers. Distrib..

[72] Carvalho S.B., Brito J.C., Crespo E.G., Watts M.E., Possingham H.P. (2011). Conservation planning under climate change: Toward accounting for uncertainty in predicted species distributions to increase confidence in conservation investments in space and time. Biol. Conserv..

[73] Radchuk V., Kramer-Schadt S., Fickel J., Wilting A. (2019). Distributions of mammals in Southeast Asia: The role of the legacy of climate and species body mass. J. Biogeogr..

[74] Morovati M., Panahandeh M., Rousta Z., Shorakaei M.J. (2015). Habitat desirability modelling of cheetah (*Acinonyx jubatus venaticus*) using maximum entropy model in central Iran (a case study: Yazd province-Dareh Anjir wildlife refuge). Appl. Ecol. Environ. Res..

[75] Abedin I., Mukherjee T., Abedin J., Kim H.-W., Kundu S. (2024). Habitat Loss in the IUCN Extent: Climate Change-Induced Threat on the Red Goral (*Naemorhedus baileyi*) in the Temperate Mountains of South Asia. Biology.

[76] Wang F., McShea W.J., Wang D., Li S., Zhao Q., Wang H., Lu Z. (2014). Evaluating Landscape Options for Corridor Restoration between Giant Panda Reserves. PLoS ONE.

[77] McRae B.H., Dickson B.G., Keitt T.H., Shah V.B. (2008). Using Circuit Theory to Model Connectivity in Ecology, Evolution, and Conservation. Ecology.

[78] McGarigal K. (2015). FRAGSTATS Help.

[79] Sertel E., Topaloğlu R.H., Şallı B., Yay Algan I., Aksu G.A. (2018). Comparison of Landscape Metrics for Three Different Level Land Cover/Land Use Maps. ISPRS Int. J. Geo-Inf..

[80] Midha N., Mathur P.K. (2010). Assessment of forest fragmentation in the conservation priority Dudhwa landscape, India using FRAGSTATS computed class level metrics. J. Indian Soc. Remote Sens..

[81] Ripple W.J., Estes J.A., Beschta R.L., Wilmers C.C., Ritchie E.G., Hebblewhite M., Berger J., Elmhagen B., Letnic M., Nelson M.P. (2014). Status and ecological effects of the world’s largest carnivores. Science.

[82] Han J., Han F., Dunets A., Batbayar B. (2024). Mapping transboundary ecological networks for conservation in the Altai Mountains. Ecol. Indic..

[83] Macdonald D.W., Bothwell H.M., Kaszta Ż., Ash E., Bolongon G., Burnham D., Can O.E., Campos-Arceiz A., Phan C., Clements G.R. (2019). Multi-scale habitat modelling identifies spatial conservation priorities for mainland clouded leopards (*Neofelis nebulosa*). Divers. Distrib..

[84] Abedin I., Singha H., Kang H.-E., Kim H.-W., Kundu S. (2024). Forecasting Suitable Habitats of the Clouded Leopard (*Neofelis nebulosa*) in Asia: Insights into the Present and Future Climate Projections Within and Beyond Extant Boundaries. Biology.

[85] Sodhi N.S., Koh L.P., Brook B.W., Ng P.K.L. (2004). Southeast Asian biodiversity: An impending disaster. Trends Ecol. Evol..

[86] Jokinen M., Hanski I., Numminen E., Valkama J., Selonen V. (2019). Promoting species protection with predictive modelling: Effects of habitat, predators, and climate on the occurrence of the Siberian flying squirrel. Biol. Conserv..

[87] Selonen V., Hongisto K., Hänninen M., Turkia T., Korpimäki E. (2020). Weather and biotic interactions as determinants of seasonal shifts in abundance measured through nest-box occupancy in the Siberian flying squirrel. Sci. Rep..

[88] Bedoya-Canas L.E., López-Hernández F., Cortés A.J. (2024). Climate Change Responses of High-Elevation Polylepis Forests. Forests.

[89] Brunke J., Radespiel U., Russo I.R., Bruford M.W., Goossens B. (2019). Messing about on the river: The role of geographic barriers in shaping the genetic structure of Bornean small mammals in a fragmented landscape. Conserv. Genet..

[90] O’Neill A.R. (2019). Evaluating high-altitude Ramsar wetlands in the Eastern Himalayas. Glob. Ecol. Conserv..

[91] La Sorte F.A., Jetz W. (2010). Projected range contractions of montane biodiversity under global warming. Proc. R. Soc. B Biol. Sci..

[92] Chen I.C., Hill J.K., Ohlemüller R., Roy D.B., Thomas C.D. (2011). Rapid range shifts of species associated with high levels of climate warming. Science.

[93] Rowe R.J., Terry R.C. (2014). Small mammal responses to environmental change: Integrating past and present dynamics. J. Mammal..

[94] Payne B.L., Bro-Jørgensen J. (2016). Disproportionate climate-induced range loss forecast for the most threatened African antelopes. Curr. Biol..

[95] Dubos N., Montfort F., Grinand C., Nourtier M., Deso G., Probst J.M., Razafimanahaka J.H., Andriantsimanarilafy R.R., Rakotondrasoa E.F., Razafindraibe P. (2022). Are narrow-ranging species doomed to extinction? Projected dramatic decline in future climate suitability of two highly threatened species. Perspect. Ecol. Conserv..

[96] Costa-Pinto A.L., Bovendorp R.S., Heming N.M., Malhado A.C., Ladle R.J. (2024). Where could they go? Potential distribution of small mammals in the Caatinga under climate change scenarios. J. Arid Environ..

[97] Lindenmayer D. (2019). Small patches make critical contributions to biodiversity conservation. Proc. Natl. Acad. Sci. USA.

[98] Wintle B.A., Kujala H., Whitehead A., Cameron A., Veloz S., Kukkala A., Moilanen A., Gordon A., Lentini P.E., Cadenhead N.C.R. (2019). Global synthesis of conservation studies reveals the importance of small habitat patches for biodiversity. Proc. Natl. Acad. Sci. USA.

