# Supplementary material for: Eco-Spatial Modeling of Two Giant Flying Squirrels (Sciuridae: Petaurista): Navigating Climate Resilience and Conservation Roadmap in the Eastern Himalaya and Indo-Burma Biodiversity Hotspots
Source: Life (Basel). 2025 Apr 3;15(4):589. doi: 10.3390/life15040589 (PMC12028898; doi:10.3390/life15040589)
Supplement: Supplementary file 1 [file life-15-00589-s001.zip › life-3528604-supplementary.pdf]

## Supplementary Materials

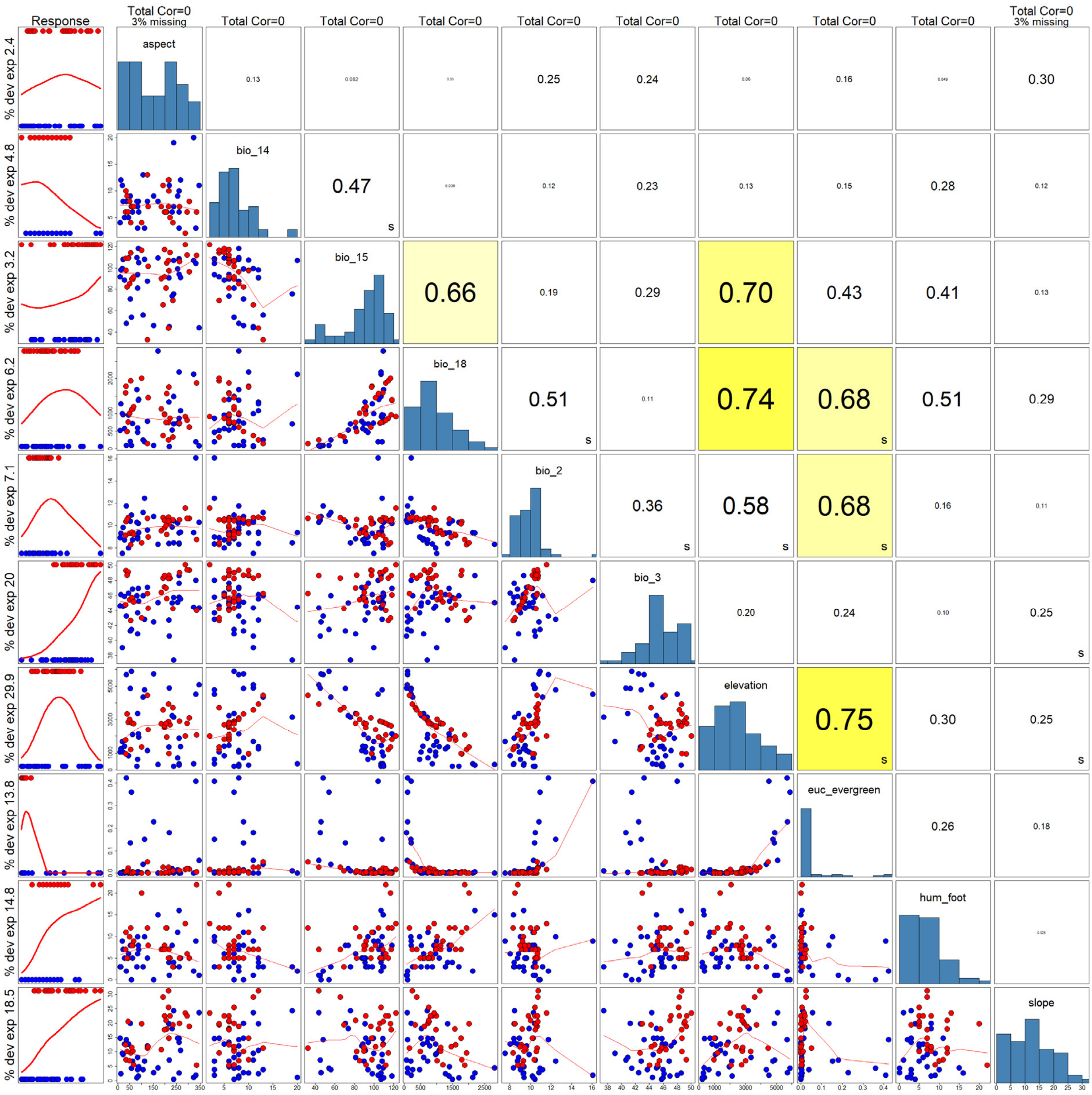


**Figure S1.** Correlations between the covariates chosen for the habitat–climate model for *P. magnificus*. The Pearson correlation coefficient is primarily used here. However, where the Spearman or Kendall correlation coefficient exceeds the Pearson correlation coefficient, an "s" or "k" is displayed in the bottom-right corner of the variable box.

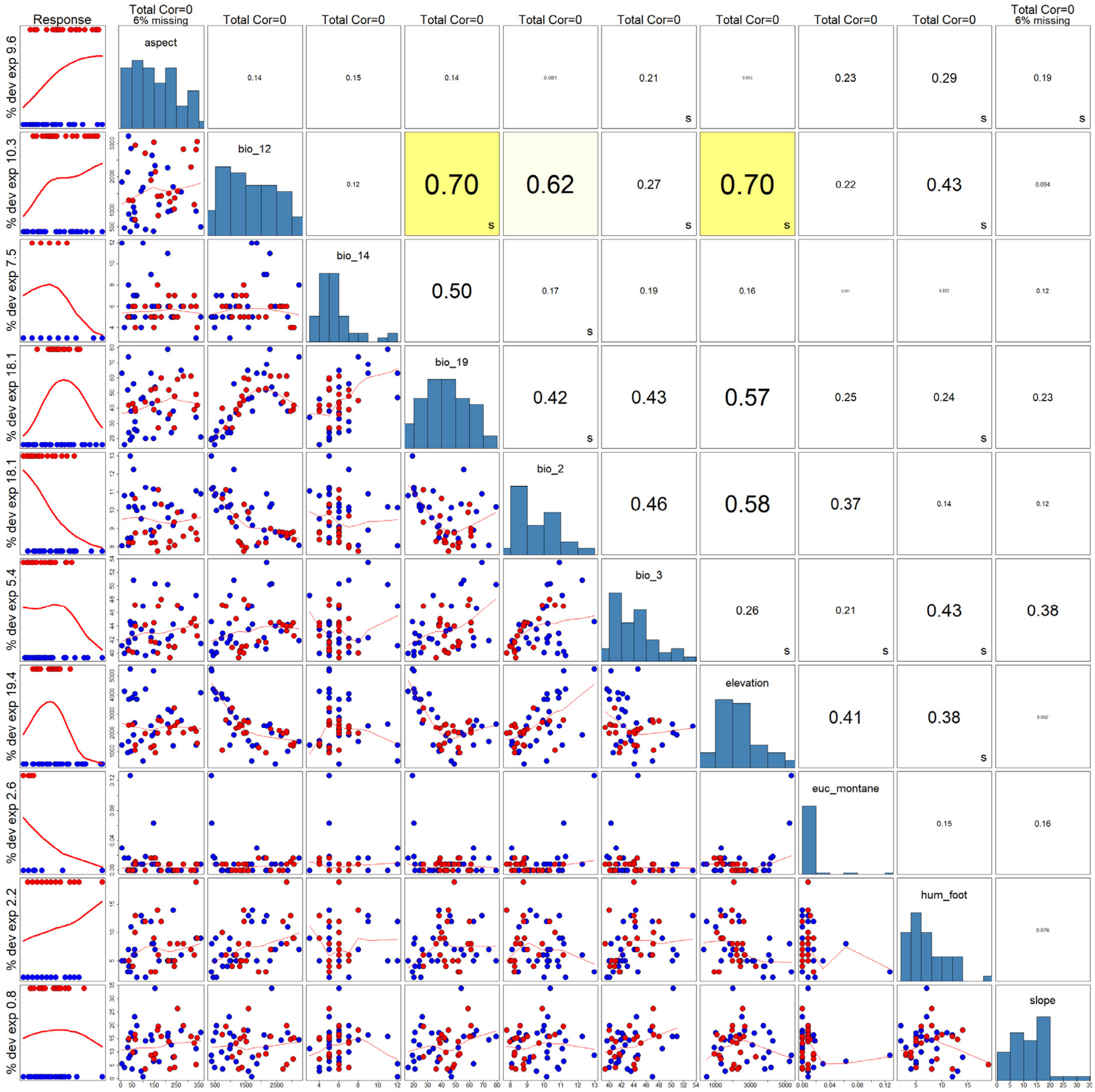


**Figure S2.** Correlations between the covariates chosen for the habitat–climate model for *P. nobilis*. The Pearson correlation coefficient is primarily used here. However, where the Spearman or Kendall correlation coefficient exceeds the Pearson correlation coefficient, an "s" or "k" is displayed in the bottom-right corner of the variable box.

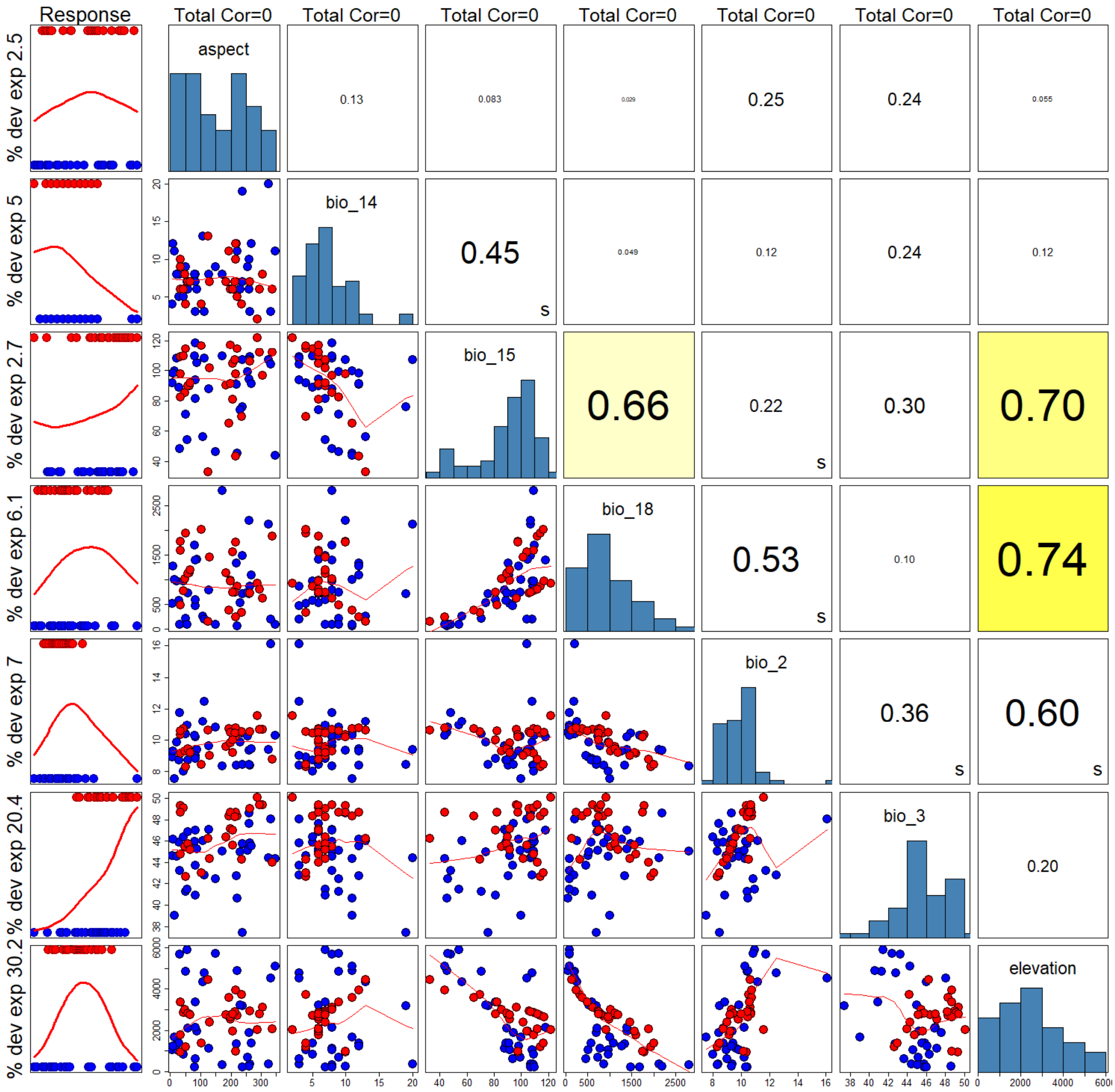


**Figure S3.** Correlations between the covariates chosen for the climate-only model for *P. magnificus*. The Pearson correlation coefficient is primarily used here. However, where the Spearman or Kendall correlation coefficient exceeds the Pearson correlation coefficient, an "s" or "k" is displayed in the bottom-right corner of the variable box.

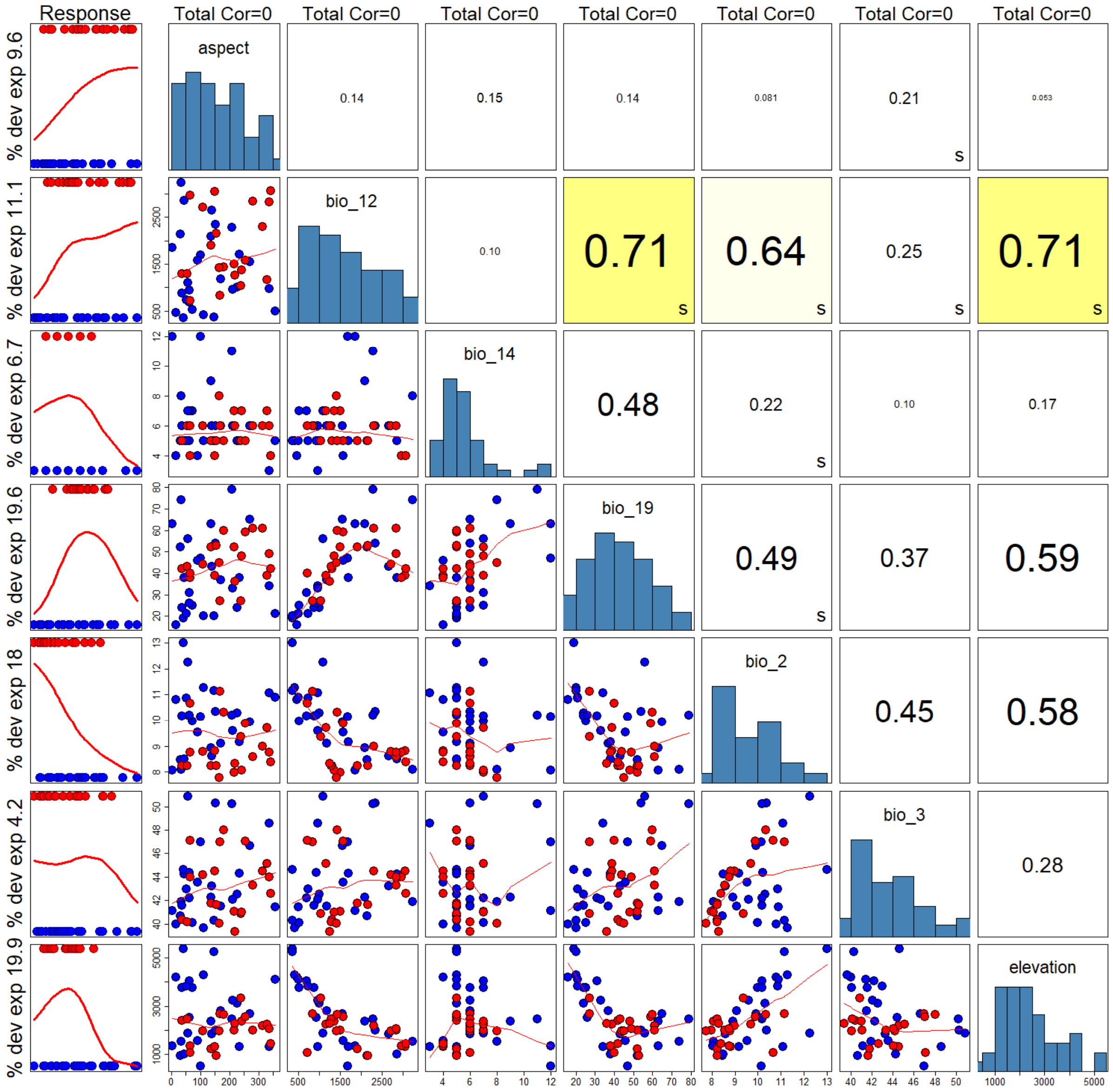


**Figure S4.** Correlations between the covariates chosen for the climate-only model for *P. nobilis*. The Pearson correlation coefficient is primarily used here. However, where the Spearman or Kendall correlation coefficient exceeds the Pearson correlation coefficient, an “s” or “k” is displayed in the bottom-right corner of the variable box.

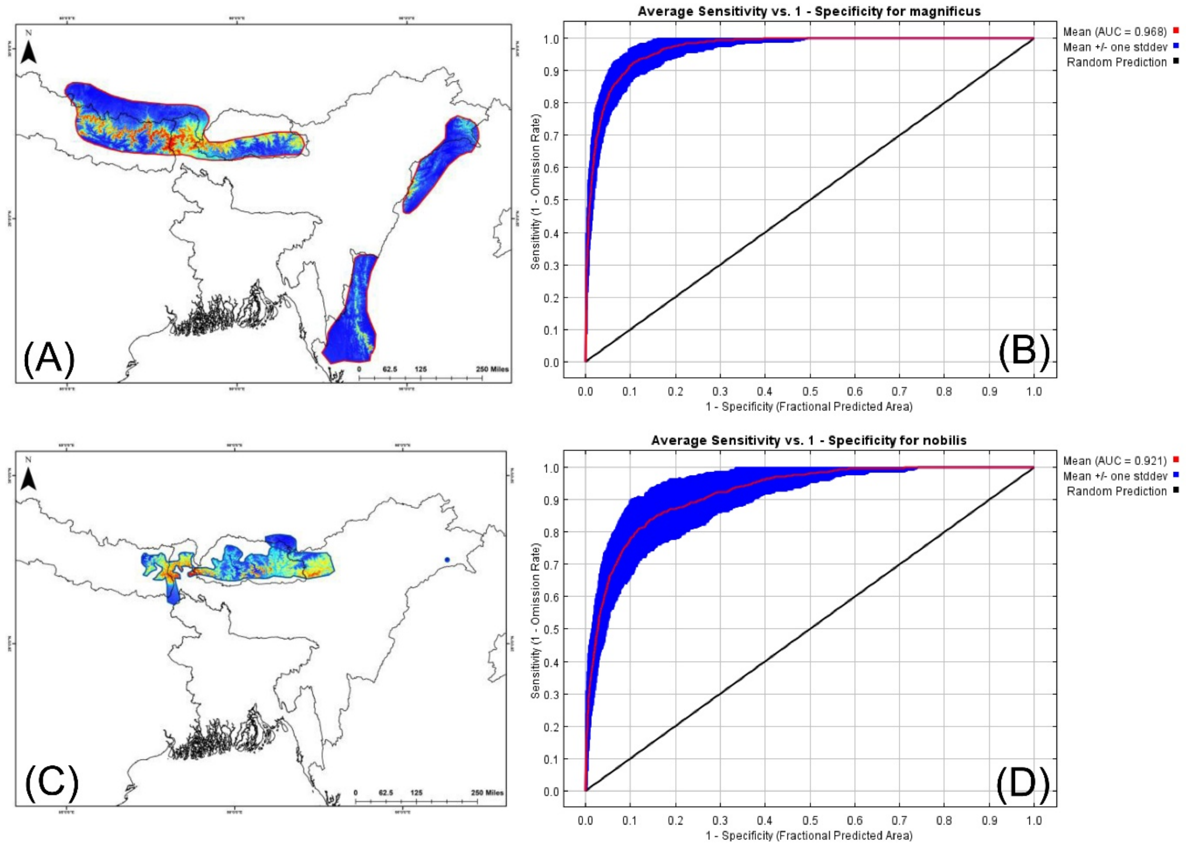


**Figure S5.** Climate-only modeling approach for the two Petaurista species. (A) Habitat suitability maps and (B) receiver operating characteristic (ROC) curves and area under the curve (AUC) evaluation plot for *P. magnificus*. (C) Habitat suitability maps and (D) receiver operating charac-teristic (ROC) curves and area under the curve (AUC) evaluation plot for *P. nobilis*.

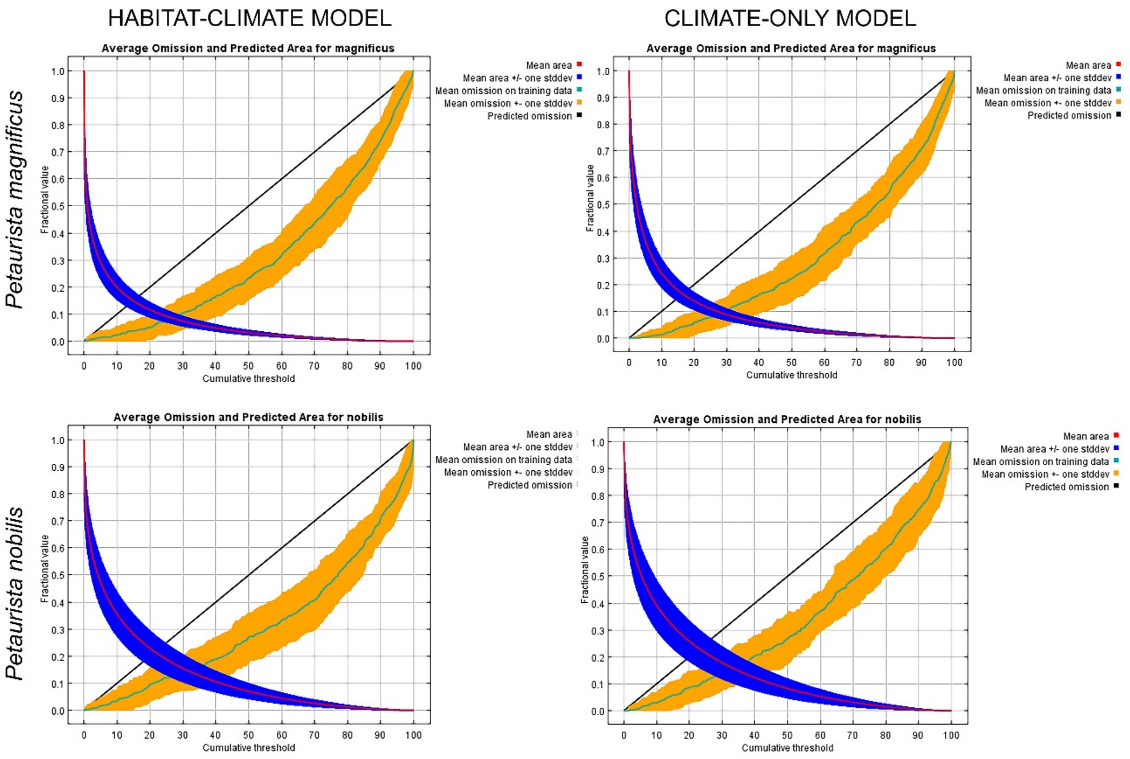


**Figure S6.** Curves showing the training omission rate and predicted area as a function of the cumulative threshold, averaged over the replicate runs for the two *Petaurista* species under two different modeling approaches.

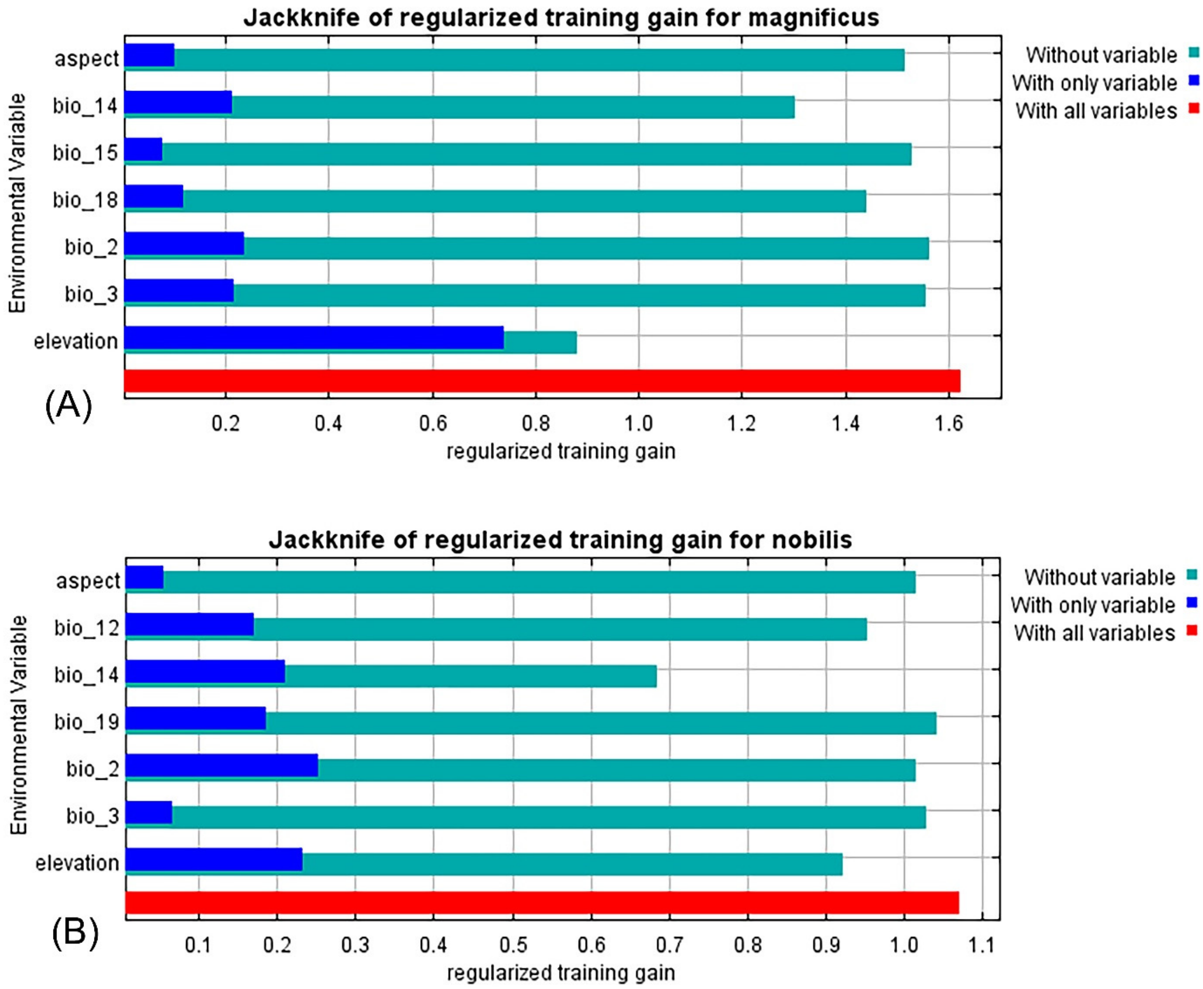


**Figure S7.** Jackknife test for all the selected variables in the climate-only model, where the blue bar shows the importance of each variable in explaining the data variation where used separately. The green bar shows the loss in overall gain after the particular variable was dropped. Red bar = total model gain. (A) *P. magnificus* and (B) *P. nobilis*.

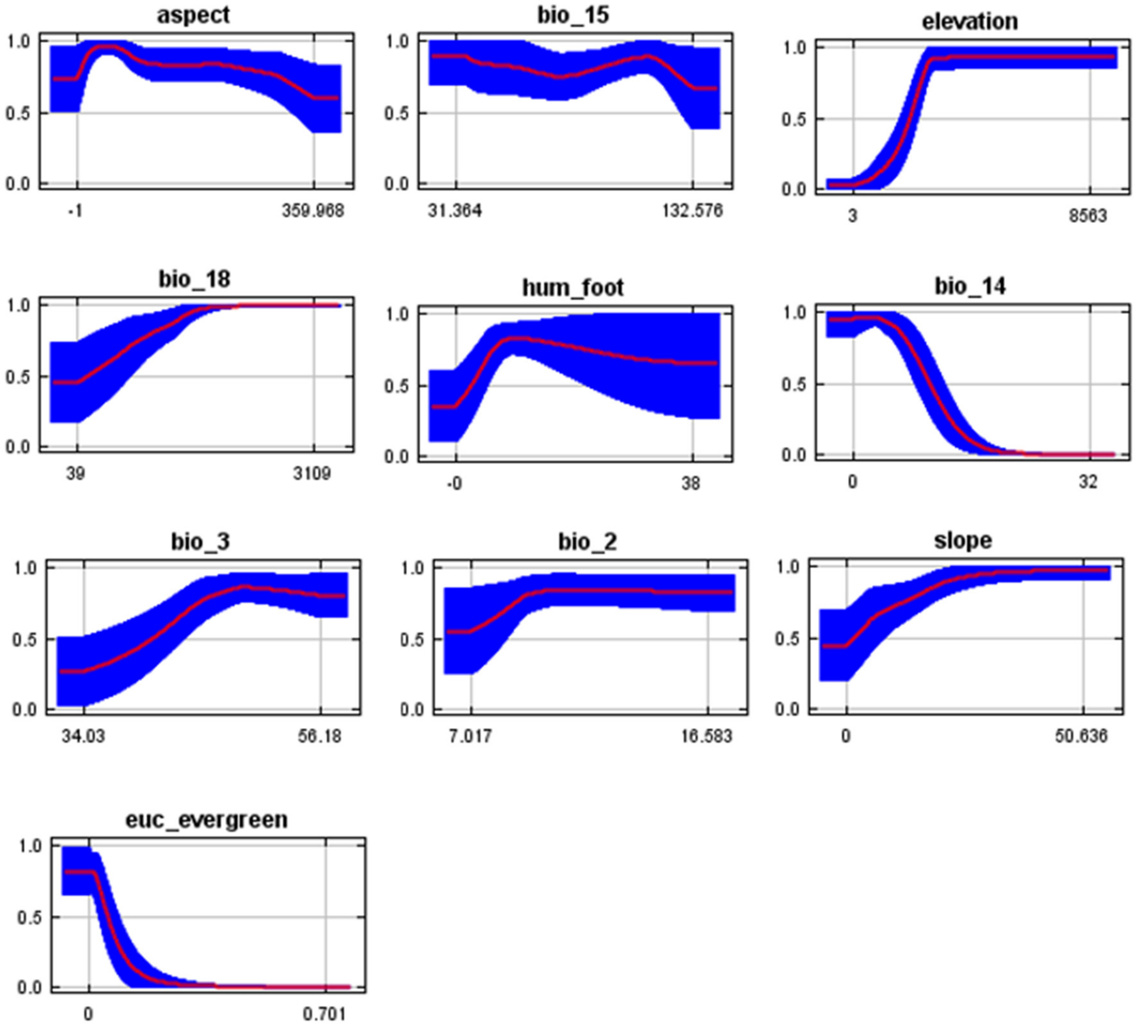


**Figure S8.** Curves showing how each environmental variable affects the Maxent prediction for *P. magnificus* in the habitat–climate model; how the predicted probability of presence changes as each environmental variable is varied, keeping all other environmental variables at their average sample value; the marginal effect of changing exactly one variable, whereas the model may take advantage of sets of variables changing together; and the mean response of the 50 replicate Maxent runs (red) and the mean +/− one standard deviation (blue, two shades for categorical variables).

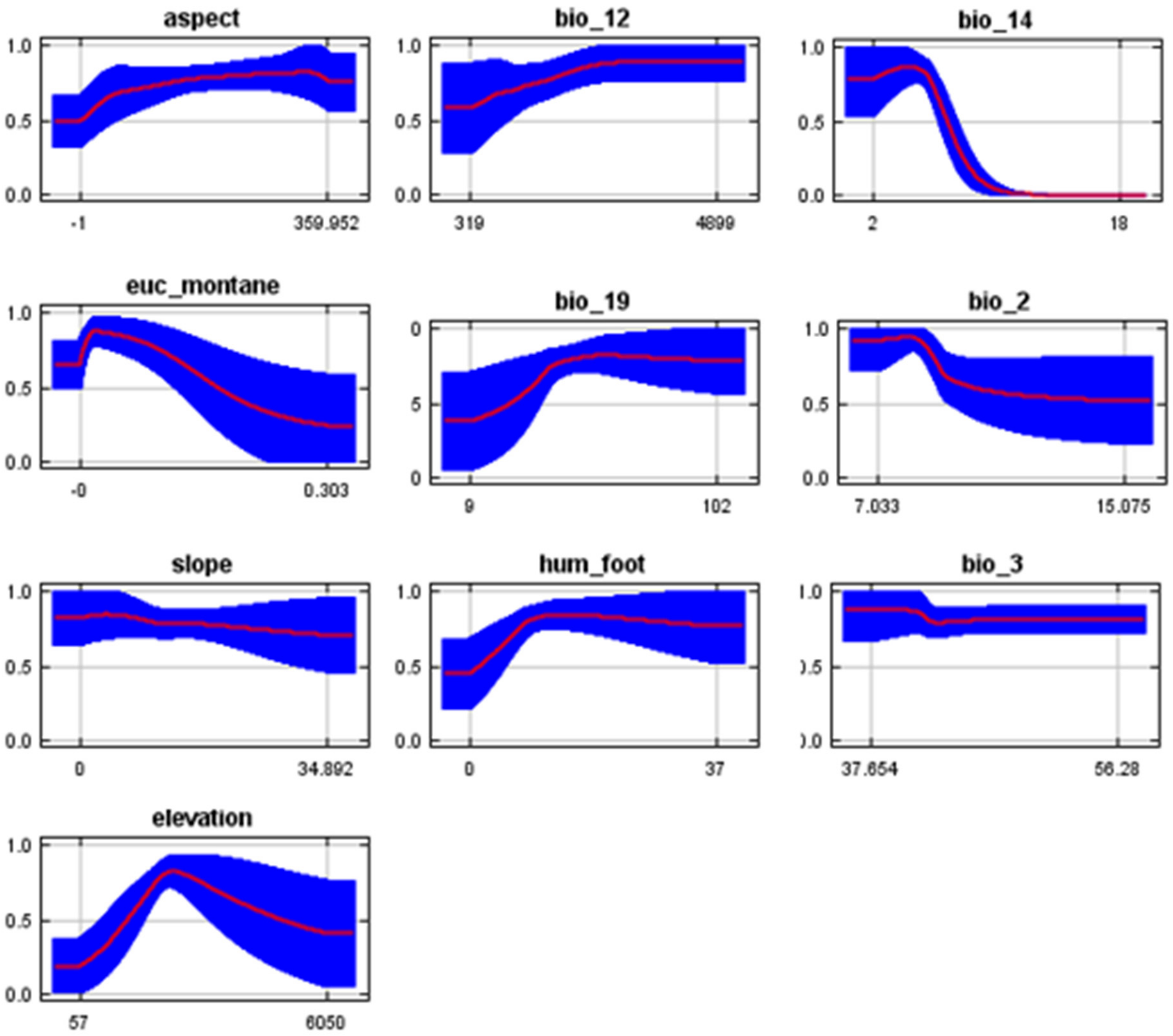


**Figure S9.** Curves showing how each environmental variable affects the Maxent prediction for *P. nobilis* in the habitat–climate model; how the predicted probability of presence changes as each environmental variable is varied, keeping all other environmental variables at their average sample value; the marginal effect of changing exactly one variable, whereas the model may take advantage of sets of variables changing together; and the mean response of the 50 replicate Maxent runs (red) and the mean +/− one standard deviation (blue, two shades for categorical variables).

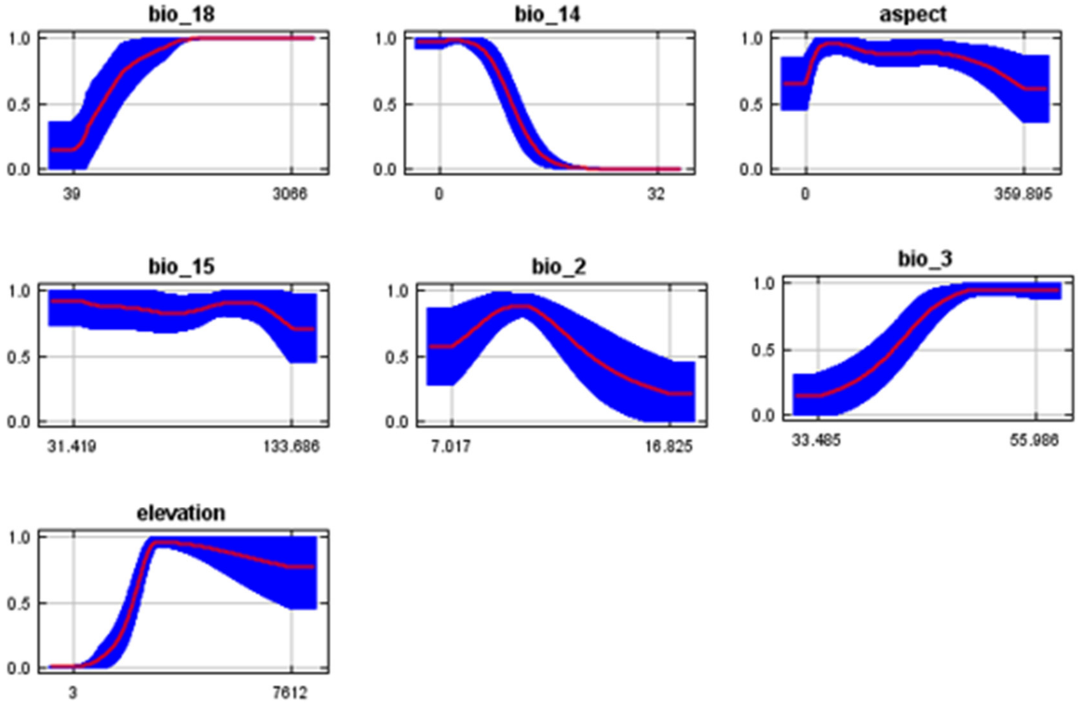


**Figure S10.** Curves showing how each environmental variable affects the Maxent prediction for *P. magnificus* in Climate-only Model. The curves show how the predicted probability of presence changes as each environmental variable is varied, keeping all other environmental variables at their average sample value. The curves show the marginal effect of changing exactly one variable, whereas the model may take advantage of sets of variables changing together. The curves show the mean response of the 50 replicate Maxent runs (red) and and the mean +/- one standard deviation (blue, two shades for categorical variables).

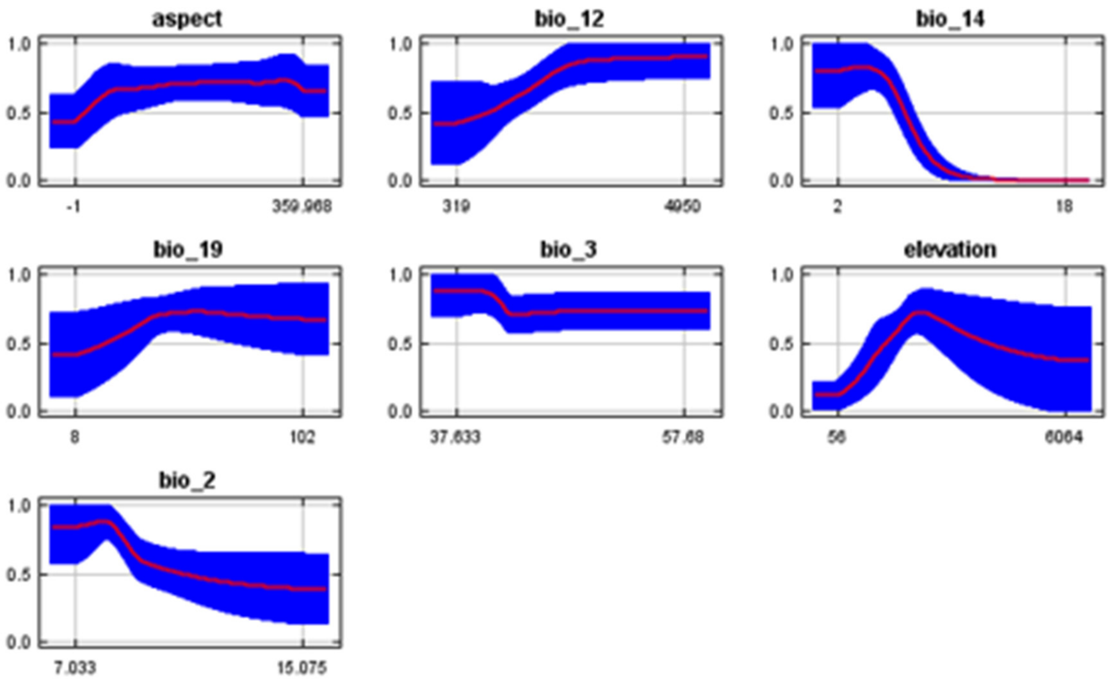


**Figure S11.** Curves showing how each environmental variable affects the Maxent prediction *for P. nobilis* in the climate-only model; how the predicted probability of presence changes as each environmental variable is varied, keeping all other environmental variables at their average sample value; the marginal effect of changing exactly one variable, whereas the model may take advantage of sets of variables changing together; and the mean response of the 50 replicate Maxent runs (red) and the mean +/− one standard deviation (blue, two shades for categorical variables).

**Table S1.** Locality information on *P. magnificus* and *P. nobilis* specimens archived at the National Zoological Collections of the Zoological Survey of India, Kolkata, India.

| ZSI Voucher No. | Species | Sex | Locality details mentioned in the skin tags |
|---|---|---|---|
| 7551 | *Petaurista magnificus* (Hodgson, 1836) | Female | Not available |
| 9728 | *Petaurista magnificus* (Hodgson, 1836) | - | Nepal |
| 9729 | *Petaurista magnificus* (Hodgson, 1836) | - | Sikkim |
| 9730 | *Petaurista magnificus* (Hodgson, 1836) | Juvenile | Sikkim |
| 9732 | *Petaurista magnificus* (Hodgson, 1836) | - | Darjeeling, West Bengal |
| 9733 | *Petaurista magnificus* (Hodgson, 1836) | - | Not available |
| 24276 | *Petaurista nobilis* (J. E. Gray, 1842) | Male | Tumin (1475 mt) East district, Sikkim |
| 24277 | *Petaurista nobilis* (J. E. Gray, 1842) | Male | Demthang, South Sikkim |
| 23262 | *Petaurista nobilis* (J. E. Gray, 1842) | Female | Selimbong (2286 mt), Darjeeling, West Bengal |
| 24274 | *Petaurista nobilis* (J. E. Gray, 1842) | Female | Demthang, South Sikkim |
| 24275 | *Petaurista nobilis* (J. E. Gray, 1842) | Male | Ralang, South Sikkim |
| 23260 | *Petaurista nobilis* (J. E. Gray, 1842) | Female | Ghoombhanjan, Darjeeling, West Bengal |
| 23261 | *Petaurista nobilis* (J. E. Gray, 1842) | Male | Ghoombhanjan, Darjeeling, West Bengal |
| 23254 | *Petaurista nobilis* (J. E. Gray, 1842) | Female | Ghoombhanjan, Darjeeling,West Bengal |
| 26762 | *Petaurista nobilis* (J. E. Gray, 1842) | Female | Dankhasari Forest Lelock (8000 ft) Neora Valley, Darjeeling, West Bengal |
